# Dynamics simulation and autonomous driving algorithm integration of unmanned harvester based on TruckSim/Simulink

**DOI:** 10.3389/fpls.2026.1754703

**Published:** 2026-03-03

**Authors:** Liang Sun, Qiaolong Wang, ZiYang Kong, Wenfei Feng, Tao Xu, Gaohong Yu

**Affiliations:** 1 School of Mechanical Engineering, Zhejiang Sci-Tech University, Hangzhou, China; 2 Key Laboratory of Agricultural Equipment for Hilly and Mountainous Areas in Southeastern China (Co-construction by Ministry and Province), Ministry of Agriculture and Rural Affairs, Hangzhou, China; 3 Zhejiang Provincial Key Laboratory of Agricultural Intelligent Sensing and Robotics, Hangzhou, China

**Keywords:** agricultural scenes, dual heuristic search, EKF, hybrid A*, intelligent agricultural equipment, lane keeping, PID control

## Abstract

To enhance the path tracking accuracy and dynamic adaptability of small unmanned harvesters in complex farmland environments, this paper proposes a simulation and autonomous driving algorithm framework based on TruckSim and Simulink. By innovatively integrating TruckSim’s high-precision dynamic simulation with Simulink’s powerful algorithm development capabilities, we have constructed a comprehensive simulation platform that accurately models the harvester’s behavior in agricultural settings. This platform not only accurately simulates dynamic responses under various operating conditions but also facilitates efficient testing and validation of autonomous driving algorithms, thereby significantly shortening development cycles and lowering field-testing costs. For path planning, we implement a hybrid A* algorithm with dual heuristic search strategy to generate optimal paths in typical static agricultural operations. At the control level, a PID controller is designed to optimize path tracking and speed control performance. Furthermore, an Extended Kalman Filter-based road adhesion coefficient identification method is introduced, which integrates multi-sensor data to dynamically estimate road conditions and adjust control strategies accordingly. To enhance system robustness, a PID-based lane-keeping algorithm with steering-speed coordination mechanism is incorporated, significantly improving operational stability in various farmland environments. Field validation results demonstrate that this research provides an innovative simulation tool and effective algorithm validation platform, advancing the development of intelligent agricultural equipment.

## 1 Introduction

Autonomous driving technology for unmanned harvesters has become a focal point in the field of agricultural automation (Fujinaga, 2025). Developing efficient and precise autonomous driving technology has become key to improving the work efficiency and stability of agricultural machinery (Bai et al., 2023). However, existing technologies still encounter bottlenecks in environmental perception, path planning, and control strategy optimization during operation, especially in complex farmland environments, posing severe challenges to the stability and robustness of autonomous driving systems.

In response to these challenges, extensive research both domestically and internationally has explored various issues related to agricultural autonomous driving technology, with most studies focusing on path planning and environmental perception. International research has utilized deep learning and computer vision technologies to achieve obstacle detection and recognition in farmland environments, performing instance segmentation of crops, obstacles, and field roads through visual sensors. For example, vision perception systems based on convolutional neural networks (CNN) and deep learning have been widely applied in agricultural robots to enable precise detection and identification of plants, weeds, and crops (Indolia et al., 2018). In terms of path planning, foreign researchers often employ traditional algorithms such as A* and Dijkstra to search for the optimal path from start to end points in complex farmland (Rachmawati and Gustin, 2020). At the same time, deep learning methods like CNNs and long short-term memory networks (LSTM) have also been introduced to enhance the intelligence and adaptability of path planning. Additionally, environmental perception technologies based on LiDAR and RGB cameras combined with machine learning methods have significantly improved the efficiency and accuracy of path planning, while also enhancing the stability and precision of autonomous navigation systems.

Currently, many agricultural autonomous driving systems can achieve efficient path planning in static environments, but their performance is suboptimal in dynamic and complex obstacle scenarios. In recent years, deep reinforcement learning (DRL) has emerged as a new direction for path planning. This approach enables agricultural robots to autonomously learn and optimize strategies, allowing them to self-adjust in dynamic environments and enhancing the intelligence and adaptability of path planning (Li et al., 2024). With advancements in deep learning and sensor technologies, multi-sensor fusion methods for environmental perception have made significant progress. For example, systems combining LiDAR and visual sensors can more accurately identify obstacles and crops in farmland, enabling real-time path planning and control decisions based on this information (Nehme et al., 2021; Ban et al., 2024) proposed a method (named CLI-Fusion) based on camera fusion, light detection and ranging (LiDAR), and an inertial measurement unit (IMU) to accurately extract real-time navigation lines between maize crop rows during the seedling stage (Kang et al., 2022) proposed a LiDAR-camera fusion visual system based on LiDAR and a fruit segmentation method utilizing deep learning to achieve accurate fruit localization within natural orchard environments.

In terms of control strategies, PID controllers have found extensive application in autonomous driving and agricultural machinery control due to their simplicity, ease of implementation, and robust performance (Peicheng et al., 2022). For instance, in vehicle path tracking control, PID controllers achieve precise guidance by adjusting lateral deviation and heading deviation; in speed control, they effectively suppress external disturbances to maintain stable vehicle velocity (Kebbati et al., 2021). Research both domestically and internationally indicates that PID controllers demonstrate excellent stability and responsiveness within agricultural machinery control systems, with their advantages particularly evident when handling the non-linear and dynamic variations inherent in field environments (Jin X. et al., 2024). For instance, certain studies integrate PID controllers with visual navigation systems to achieve autonomous navigation and stable control for tractors during field operations (Bai et al., 2023). Other research utilizes PID controllers to regulate the travel speed and header height of combine harvesters, thereby enhancing operational quality and efficiency (Zhang et al., 2025). These application cases validate the practicality and reliability of PID controllers within agricultural autonomous driving systems.

In practical applications, sensor noise and uncertainties affect system accuracy. Therefore, research has begun exploring filtering algorithms such as Kalman filtering and extended Kalman filtering to improve data quality and accuracy. By fusing multi-sensor data (such as wheel speed sensors, accelerometers, and gyroscopes) (Chua et al., 2011), the system can estimate the road adhesion coefficient in real time, dynamically adjust control strategies, and optimize path tracking and speed control. The road adhesion coefficient is crucial for the traction and stability of agricultural vehicles; its variations under different terrains and weather conditions significantly impact vehicle stability and safety. The PID controller adjusts vehicle path tracking and lateral stability in real time, ensuring automatic correction of deviations and maintaining stable driving across various farmland environments. By continuously optimizing the proportional, integral, and derivative parameters, the control strategy maintains high stability and accuracy under changing conditions. This innovative technology enables the simulation system to more realistically reflect actual control requirements and provides an effective means to test the robustness of autonomous driving algorithms in dynamic environments, further enhancing the adaptability and reliability of agricultural autonomous driving systems.

In order to achieve the vehicle’s dynamic response under different operating conditions, conduct real-time testing and validation of autonomous driving algorithms, shorten the algorithm development cycle, reduce the cost of field testing, improve research and development efficiency, and lower development costs, this study provides practical solutions. The main work of this research is as follows:

1. Develop a high-fidelity simulation platform by innovatively adopting a TruckSim-Simulink collaborative framework to establish a wheeled equivalent model of a tracked harvester, achieving high-precision simulation of key dynamic characteristics such as yaw response and lateral acceleration.
2. A multimodal environmental perception system is proposed, utilizing a classic PID controller to optimize path tracking and speed control, and introducing an Extended Kalman Filter (EKF) for rapid identification of road adhesion coefficients.
3. Proposed an adaptive decision-making and planning framework that uses a hybrid A* algorithm for path planning. By combining a dual heuristic search strategy, it can quickly find the optimal path and improve search efficiency.

In summary, this study proposes an integrated simulation and control framework based on TruckSim/Simulink to enhance the autonomous operation capabilities of small unmanned harvesters in complex agricultural environments. By constructing a high-fidelity collaborative simulation platform, the framework resolves the challenge of lacking dedicated models for target harvesters; innovatively integrates extended Kalman filtering for real-time identification of road adhesion coefficients, providing critical environmental perception data for control decisions; and employs a hybrid A* algorithm with PID control to achieve efficient path planning and precise tracking. The value of this research lies not only in algorithmic integration and methodological innovation, but also in its provision of practical technical support for smart agriculture development: the constructed simulation platform can significantly shorten the R&D cycle of autonomous driving systems for agricultural equipment while reducing field testing costs and risks; The proposed perception and control strategies markedly enhance the harvester’s operational adaptability and safety across complex terrains such as hilly regions and slippery, muddy fields; The entire system establishes a critical technological foundation for advancing from single-machine intelligent operations to future multi-machine collaborative operations in unmanned farms. It holds clear engineering application prospects for addressing agricultural labor shortages and driving the transformation of agricultural production methods towards precision and automation (Montemerlo et al., 2008).

## 2 Materials and methods

### 2.1 Establishment of the dynamic model

This study aims to develop an autonomous driving algorithm simulation framework for small unmanned harvesters. However, due to the lack of a high-fidelity specialized model precisely matching the target platform—a small full-feed tracked combine harvester—in the standard model library of the selected simulation software (TruckSim), this study strategically selected the built-in TruckSim model (used for theoretical model derivation in Section 1 of this chapter) and the Suzhou King Long KLQ6125D bus model (used for vehicle dynamics simulation and control system validation in Section 4 of this chapter) as alternative simulation platforms (Mechanical Simulation Corporation, 2021). This choice is based on the following considerations:

#### 2.1.1 Universality of dynamic principles

The fundamental physical principles involved in core research topics such as vehicle yaw stability, lateral dynamics, tire-road interaction (adhesion coefficient identification), and path tracking control (PID)—including Newtonian mechanics, kinematics, and control theory—are universally applicable to wheeled vehicles. Both the tractor-trailer model, with its structural complexity (multi-body connections), and the bus model, in terms of maneuvering stability analysis, can effectively support this study’s validation requirements for core algorithms (A*/hybrid A*, EKF, PID).

#### 2.1.2 Parameter adjustability

TruckSim allows for extensive adjustments to key parameters of the selected model (such as mass, wheelbase, track width, moment of inertia, tire characteristics, etc.). In this study, the simulation model was specifically parameterized based on publicly available data and the key characteristic parameters of small harvesters (such as curb weight range, typical dimensions, and converting tracked ground contact characteristics into equivalent wheeled parameters), aiming to accurately replicate the typical features of the target harvester at the core dynamic behavior level (including yaw response, lateral acceleration, and path-following capability).

#### 2.1.3 Consideration of track characteristics

The target harvester is a tracked vehicle, which inherently differs from the selected wheeled model (e.g., steering mechanism, ground pressure distribution, slip characteristics). In this study, tire model parameters (such as longitudinal slip stiffness and cornering stiffness) are adjusted to approximate the equivalent behavior of the tracked vehicle in path tracking and stability control. This simplification is reasonable and effective for validating the core control algorithms under low-speed, small-to-medium steering angle conditions (typical speeds for field operations:<2.0 m/s). Track-specific dynamic effects (such as steering resistance torque and ground deformation) will be the focus of investigation in real vehicle testing.

To ensure the selected wheeled model is dynamically equivalent to the target tracked harvester, this study conducted systematic parameter mapping and response comparison analysis, with the following specific quantitative metrics:

##### 2.1.3.1 Equivalent adjustment of mass-inertia parameters

The target harvester’s total mass is m_h_=1200kg. The moment of inertia about the Z-axis, *I_z,h_*, is estimated at approximately 1800 kg·m² (based on empirical formulas for external dimensions and mass distribution). The selected bus model (KLQ6125D) has an original kerb weight of 5025 kg, with *Iz* approximately 12000 kg·m². Using Trucksim’s parameter adjustment function, the simulation model’s mass was proportionally scaled to 1250 kg (error +4.2%) and Iz adjusted to 1950 kg·m²(error +8.3%) to ensure the lateral moment of inertia remained within a reasonable range equivalent to the target harvester.

##### 2.1.3.2 Geometric dimensions and kinematic parameters mapping

The track gauge of the harvester is 710 mm, with both front and rear wheelbases of the wheeled model set to 700 mm (error -1.4%). By adjusting the wheelbase to 2200 mm and positioning the center of gravity at the wheelbase midpoint, steady-state yaw rate response during low-speed steering aligns with that of tracked vehicles. Verification through step-turn simulations at *v_x_* = 1.0 m/s and front wheel angle δ=10° demonstrated steady-state yaw rates of 0.152 rad/s for the selected model and 0.165 rad/s for the target harvester’s simplified model, yielding a relative error of 7.9%.

##### 2.1.3.3 Equivalent validation of tyre/track force characteristics

To simulate the ground pressure distribution and force transmission characteristics of the track, adjustments were made to the longitudinal stiffness *C_x_* and lateral stiffness *C_α_* based on the Magic Formula tyre model: *C_x_*, *C_α_* were set to *C_x_* = 1.2×10^5^N, *C_α_* = 8×10^4^N/rad. This ensures consistency with the track traction-slip rate curve trend within the low-speed slip range (<0.1). In simulations at *v_x_* = 1.5 m/s with sinusoidal steering input (amplitude 8°, frequency 0.2 Hz), the normalized root mean square error (NRMSE) between the simulated lateral acceleration response amplitude and the expected value for the tracked vehicle was 13.2%.

##### 2.1.3.4 Path tracing performance consistency verification

Under identical PID control parameters (Kp=1.2, Ki=0.05, Kd=0.12), linear tracking and S-curve tracking simulations were conducted to compare the selected model with the simplified target harvester model. Results indicate: During straight-line tracking (speed 1.0 m/s), the mean lateral errors were 0.041 m (wheeled) and 0.038 m (tracked), representing a 7.9% difference. During S-curve tracking, the maximum lateral errors were 0.118 m and 0.125 m respectively, differing by 5.6%.

##### 2.1.3.5 Equivalence comprehensive evaluation indicators

Define the equivalence evaluation function, whose expression is shown in Equation 1:

(1) $$\begin{array}{c} E_{eq}=\frac{1}{n}\sum_{i=1}^{n}\left|\frac{y_{wheel,i}-y_{track,i}}{y_{track,i}}\right|\times 100\% \end{array}$$

Among these, y encompasses key response variables such as the peak yaw rate, lateral acceleration amplitude, and path tracking error. Based on simulations of six typical operating conditions, the average equivalent error *E_eq_* was calculated to be 11.7%, indicating that the selected wheeled model exhibits acceptable dynamic equivalence with the target tracked harvester under low-speed conditions and small-to-medium steering angles.

In summary, through the aforementioned parameter adjustments and response comparisons, the selected tractor-trailer and bus models demonstrate excellent consistency with the target harvester in terms of mass inertia, kinematic response, force characteristics, and path-following performance. This provides a reasonable and reliable simulation platform for subsequent control algorithm validation.

The following is the specific parameter adjustment table for model equivalent amplification.

The reference model established in this section primarily focuses on the vehicle’s yaw stability and lateral dynamic response, both of which form the foundation of the dynamics of wheeled and tracked ground vehicles. Concepts involved in the model, such as forces (tire lateral forces), moments, and geometric relationships (wheelbase, articulation point locations), are fundamentally applicable to analyzing the overall motion of tracked vehicles as well. Neglecting the effects of suspension as well as pitch and roll motions is reasonable for concentrating on the core objective of studying lateral path tracking and yaw stability, and this simplification is widely adopted in vehicle control research. The selected TruckSim model is the 3A Tractor with 3A Flatbed Trailer. The actual vehicle is the Nongjiapan 4LZ-1.5A full-feed tracked combine harvester produced by Xiangyuan Jinsui Harvester Manufacturing Co., Ltd. in Shuangfeng County, Hunan Province. The technical specifications of this combine harvester are detailed in **Table 1**.

**Table 1:** Technical parameters of harvester.

Item	Data	Unit
Overall Dimensions	3800×1660×2480	mm
Machine Weight	1200	kg
Header Working Width	1350	mm
Rated Feed Rate	1.5	kg/s
Operating Speed	0.6-2.0	m/s
Track Gauge	710	mm

**Figure 1:**
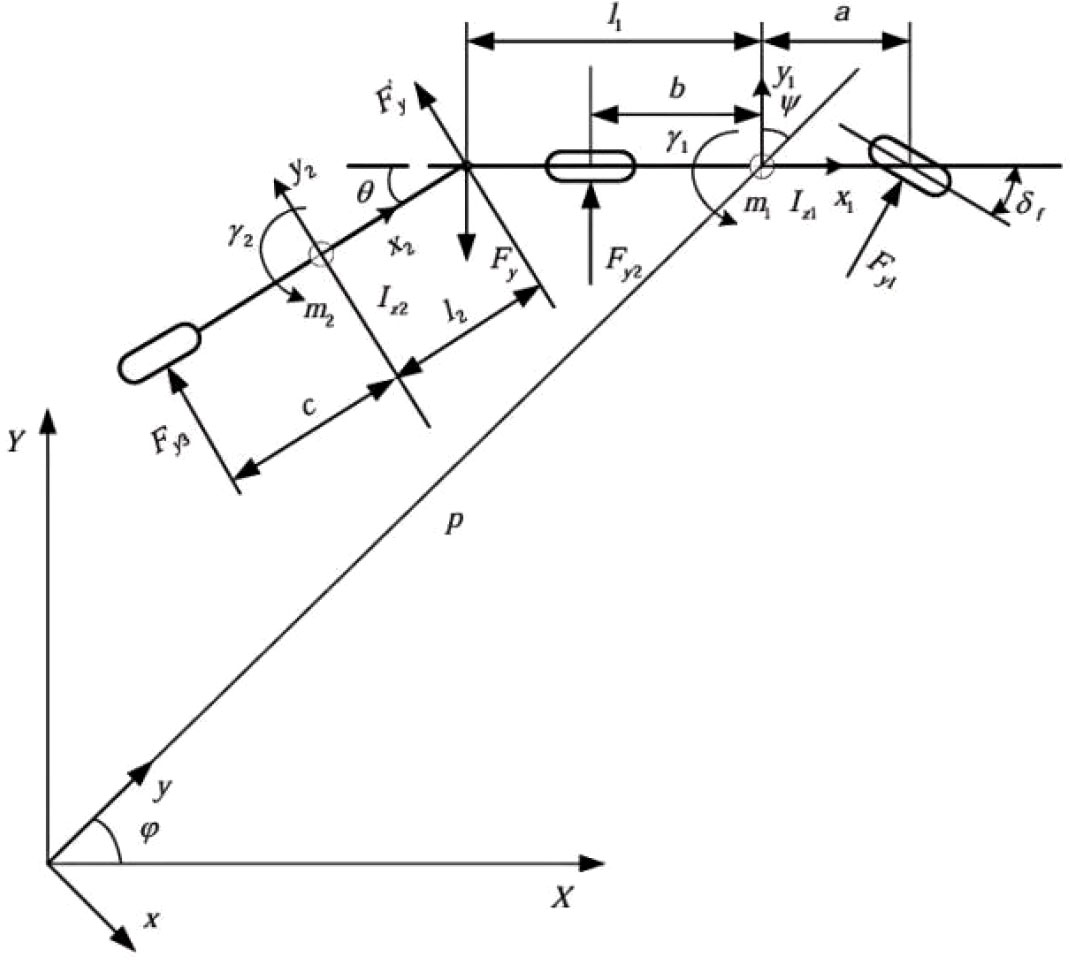
Model simplification diagram.

**Figure 2:**
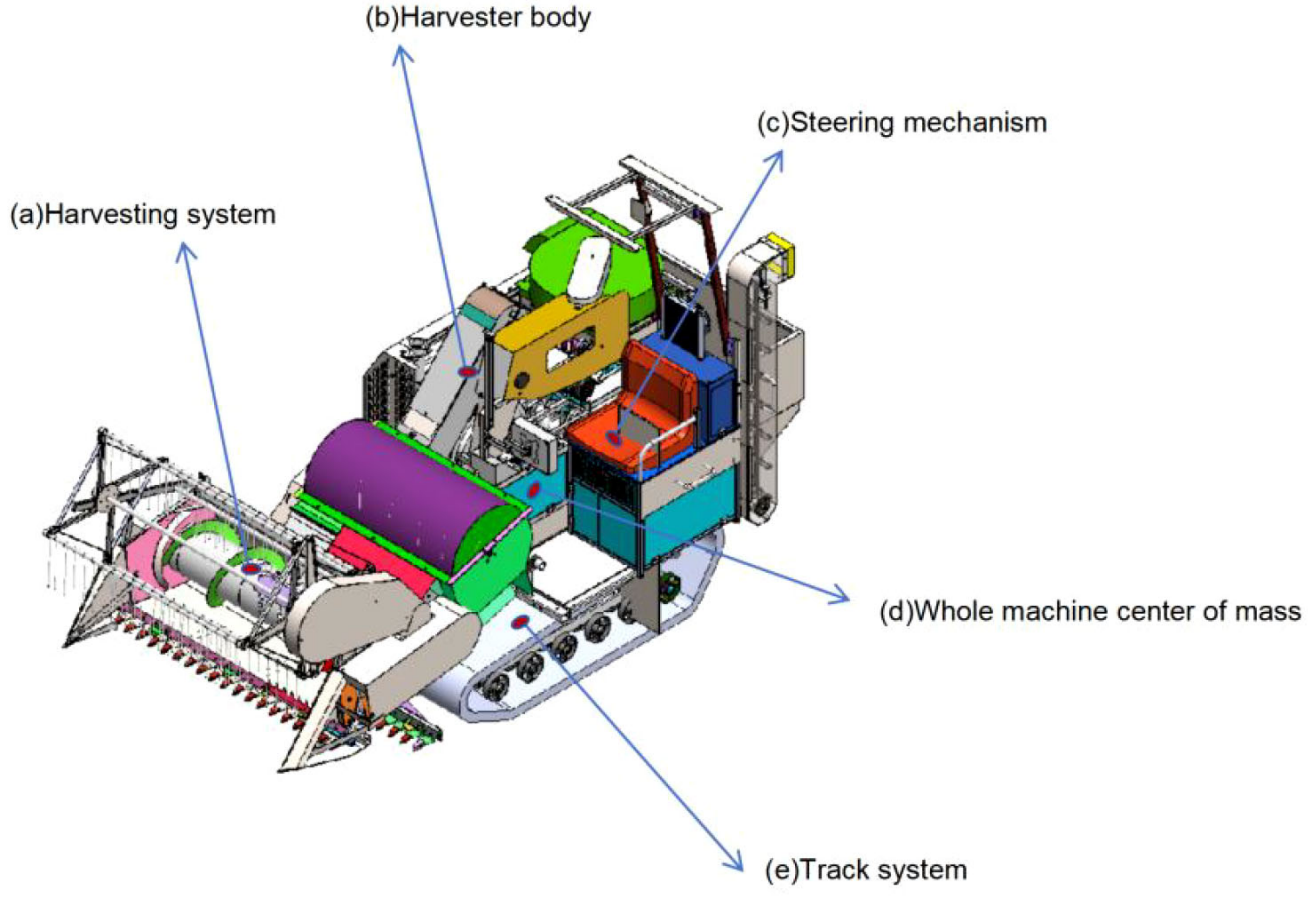
3D model of a combine harvester: **(a)** Harvesting system **(b)** Harvester body **(c)** Steering mechanism **(d)** Whole machine center of mass **(e)** Track system.

To simplify the modeling, this paper simplifies the two drive axles of the tractor into a single axle, and the three axles of the semi-trailer into a single axle, as shown in **Figure 1**. The simplified dynamic model of the tractor-semitrailer shown in this figure is an equivalent wheeled abstraction of the actual tracked harvester in **Figure 2**, used to simulate its core yaw and lateral dynamic behavior. This four-degree-of-freedom reference model can describe the yaw motion, lateral motion, and dynamic characteristics of the tractor and semi-trailer (Rajamani, 2012). Based on this, the following assumptions are made: the front wheel steering angle is taken as the input, and the influence of the steering system is neglected.

1. Assuming the vehicle’s longitudinal speed remains constant;
2. Assuming the vehicle is traveling on a flat road surface, the vertical motion of the vehicle can be neglected.
3. Ignoring pitch and roll movements;
4. Ignoring the effects of aerodynamics and road gradient;

The above assumptions are based on the actual operational characteristics of agricultural harvesters: ① The tracked harvester operates at a low speed (0.6-2.0 m/s), and longitudinal acceleration changes can be neglected; ② The slope of farmland roads is usually less than 5°, making the assumption of a level road surface reasonable (Chen et al., 2022); ③ The suspension system has a minimal effect on low-speed lateral dynamics, so pitch and roll are neglected (Sert and Boyraz, 2017); ④ Air resistance at low speeds is less than 3% of the rated tractive force (calculated using parameters in **Table 1**) and is therefore ignored. To construct the dynamic model of the tractor-trailer system, we conducted separate mechanical analyses of the tractor and semi-trailer, as shown in Equations 2–6.

For the lateral force equilibrium equation of the tractor’s center of gravity, the following Equation 2 is derived:

(2) $$\begin{array}{c} {\text{m}}_{1}a_{y1}=F_{y1}+F_{y2}-F_{y} \end{array}$$

Moment equation of the resultant forces about the center of gravity of the tractor, the following Equation 3 is derived:

(3) $$\begin{array}{c} I_{z1}{\dot{\gamma }}_{1}=F_{y1}a_{1}-F_{y2}b_{1}+F_{y1}l_{1} \end{array}$$

Lateral force equation of the center of gravity for a semi-trailer, the following Equation 4 is derived:

(4) $$\begin{array}{c} m_{1}a_{y2}=F_{y3}+F_{y1} \end{array}$$

Moment equation of the resultant force at the center of gravity of the semi-trailer, the following Equation 5 is derived:

(5) $$\begin{array}{c} I_{z2}{\dot{\gamma }}_{2}=F_{y}l_{2}-F_{y3}c \end{array}$$

In the formula: ${\text{m}}_{1}$, ${\text{m}}_{2}$: mass of the tractor/semi-trailer; $a_{1}$, $b_{1}$, $l_{1}$: distances from the tractor’s center of gravity to the front axle, rear axle, and articulation point, respectively; c, $l_{2}$: distances from the semi-trailer’s center of gravity to the rear axle and articulation point, respectively; $F_{y1}$, $F_{y2}$, $F_{y3}$: lateral forces on the front wheels, rear wheels, and semi-trailer wheels, respectively (Jin Y. et al., 2024). To accurately describe the complex kinematic coupling relationship between thetractor unit and semi-trailer in articulated vehicle systems, a decompositionexpression for the yaw angular velocity is introduced, as shown in Equation 6.

(6) $$\begin{array}{c} \{\begin{array}{c} \gamma_{1}=\dot{\varphi }+\dot{\psi }\\ \gamma_{2}=\dot{\varphi }+\dot{\psi }+\dot{\theta } \end{array} \end{array}$$

Among them, $\gamma_{1\;}$, $\;\gamma_{2\;}$: the yaw angular velocity (°)/s of the tractor and semi-trailer; $\dot{\varphi \;}$: the rotational angular velocity of the tractor around its own center of mass; $\dot{\;\psi }$: the coupled angular velocity caused by the articulation point; $\dot{\theta }$: he relative angular velocity of the semi-trailer relative to the tractor. This system of equations forms the basis for deriving the moment equilibrium Equations 2–5.

**Figure 2** is a 3D model of the small harvester. The simplified model in **Figure 1** is related to the actual harvester in **Figure 2** as follows: the tractor section corresponds to the main body of the harvester (including the power system and the cab); the semi-trailer section corresponds to the cutter bar and the collection system; the articulation point simulates the dynamic effects of the harvester’s steering mechanism. The core purpose of this equivalent model is to accurately replicate the typical dynamic characteristics of the target harvester in path tracking and yaw stability analysis.

The harvesting system is divided into five parts: the harvesting system, the harvester body, the steering mechanism, the track system, and the whole machine center of mass. The ‘semi-trailer’ part in **Figure 1** corresponds to the ‘cutting platform system’ in **Figure 2a**, and the “tractor” part in **Figure 1** corresponds to the ‘harvester body’ in **Figure 2b**. the ‘articulation point’ in **Figure 1** corresponds to the ‘steering mechanism’ in **Figure 2c**, the ‘center of gravity position’ in **Figure 1** corresponds to the ‘center of gravity position of the entire machine’ in **Figure 2d**, and the ‘front wheels/rear wheels’ in **Figure 1** correspond to the ‘track system’ in **Figure 2e**.

### 2.2 Road adhesion coefficient identification based on EKF

In the established dynamic model, the dynamic behavior of the tractor and semi-trailer is significantly influenced by the road adhesion coefficient μ. μ not only determines the vehicle’s traction force and stability limits but also directly affects path planning and control system performance. Therefore, to achieve high-performance autonomous driving, this paper employs the Extended Kalman Filter (EKF) method to identify the road adhesion coefficient in real time (Ding et al., 2022). EKF utilizes the system state equations, observation equations, and the statistical characteristics of process noise and measurement noise to perform optimal estimation, enabling accurate estimation of μ and providing critical road condition information to the control system (Bavdekar et al., 2011).

Real-time identification of μ is crucial for dynamically adjusting vehicle control strategies, especially in complex and variable farmland road conditions. For example, under low μ conditions, vehicle handling stability decreases, and the system must incorporate the real-time identified μ results to ensure driving safety through path planning (such as the hybrid A* algorithm) and control decisions (such as PID parameter tuning or speed limiting).

To balance computational complexity and algorithm feasibility verification, a nonlinear three-degree-of-freedom vehicle model is used as the basis for the EKF. The core design of the EKF relies on four types of information: state equations, observation equations, process noise statistical characteristics, and measurement noise statistical characteristics. EKF achieves local linearization of the nonlinear model by performing a first-order Taylor expansion of the nonlinear functions at the current state estimate point (neglecting higher-order terms), making it widely applicable to various nonlinear system estimation problems. The EKF-based road adhesion coefficient identification process includes the following steps:

#### 2.2.1 Establishment of the system state equation and measurement equation

The state equation and measurement equation reflect the dynamic characteristics of the system and the feedback information from the sensors, respectively. The specific expressions are as follows:

General form of the state equation for a nonlinear system (Equation 7):

(7) $$\begin{array}{c} \dot{x}\left(t\right)=f\left(x\left(t\right),u\left(t\right),w\left(t\right)\right) \end{array}$$

General form of the system observation equations (Equation 8):

(8) $$\begin{array}{c} y\left(t\right)=\left(x\left(t\right),v\left(t\right)\right) \end{array}$$

In the equation: $\dot{x}\left(t\right)\;$ represents the state variable;  u(t) represents the control variable; y(t)  represents the measurement output; w(t)  represents the system excitation noise;  v(t) represents the measurement noise; w(t),v(t) are independent white noises. Q is the covariance matrix of the system w(t) ‘s excitation noise, and R is the covariance matrix of the measurement noise v(t).

State equation based on the three-degree-of-freedom vehicle model (Equation 9):

(9) $$\begin{array}{c} \begin{array}{l} \{\begin{array}{c} \dot{\gamma }=\frac{a^{2}k_{1}+\;b^{2}k_{2}}{I_{z}v_{x}}r+\frac{ak_{1}-\;bk_{2}}{I_{z}}\beta -\frac{ak_{1}}{I_{z}}\delta \\ \dot{\beta }=\left(\frac{ak_{1}-\;bk_{2}}{mv_{x}^{2}}-1\right)r+\frac{k_{1}+\;k_{2}}{mv_{x}}\beta -\frac{k_{1}}{mv_{x}}\delta \\ {\dot{v}}_{x}=r\beta v_{x}+a_{x} \end{array} \end{array}\; \end{array}$$

Among them, a is the distance from the front axle to the center of mass, b is the distance from the rear axle to the center of mass, $\;k_{1}$ is the lateral stiffness of the front wheel, $k_{2}$ is the lateral stiffness of the rear wheel, $I_{z}$ is the moment of inertia around the z-axis of the vehicle, and m is the mass of the vehicle.

Measurement equation (lateral acceleration) (Equation 10):

(10) $$\begin{array}{c} a_{y}=\frac{ak_{1}-\;bk_{2}}{mv_{\chi }}r+\frac{k_{1}+\;k_{2}}{m}\beta -\frac{k_{1}}{m}\delta \end{array}$$

Among them: $\dot{x}\left(t\right)=\left[\begin{array}{c} \dot{r}\\ \dot{\beta }\\ {\dot{v}}_{x} \end{array}\right]$, $y\left(t\right)=a_{y}$

(2) Model Linearization

F(t),H(t) is the Jacobian matrix, which is the partial derivatives with respect to the state x(t) of nonlinearn function f(x(t),u(t),w(t))、 h(x(t),v(t)), Δt is the sampling time.

Define the system Jacobian matrix for the purpose of performing local linearisation of the nonlinear system. Define the system Jacobian matrix for the local linearisation of nonlinear systems, as shown in Equation (11).

(11) $$\begin{array}{c} F\left(t\right)=\left[\begin{array}{cc} \frac{\partial f_{1}}{\partial x_{1}} & \frac{\partial f_{1}}{\partial x_{m}}\\ \vdots & \vdots \\ \vdots & \vdots \\ \frac{\partial f_{m}}{\partial x_{1}} & \frac{\partial f_{m}}{\partial x_{m}} \end{array}\right],H\left(t\right)=\left[\begin{array}{cc} \frac{\partial h_{1}}{\partial x_{1}} & \frac{\partial h_{1}}{\partial x_{m}}\\ \vdots & \vdots \\ \vdots & \vdots \\ \frac{\partial h_{m}}{\partial x_{1}} & \frac{\partial h_{m}}{\partial x_{m}} \end{array}\right] \end{array}$$

Compute the discrete-time state transition matrix to discretise the continuous-time system for digital implementation, as shown in Equation 12.

(12) $$\begin{array}{c} \phi \left(t\right)=e^{F\left(t\right)*\Delta t}\approx I+F\left(t\right)*\Delta t \end{array}$$

Combining the above vehicle state-space equations and performing linearization, the matrix F(t) and H(t) can be obtained.

Derive the specific expression for the system matrix F(t), revealing the dynamic coupling relationships between the state variables. The specific formula is shown in Equation 13.

(13) $$F\left(t\right)=\left[\begin{array}{c} \frac{a^{2}r\;+\;b^{2}\beta }{I_{z}v_{x}}\\ \frac{\left(ak_{1}-\;bk_{2}-\;mv_{x}^{2}\right)}{mv_{x}^{2}}\\ \beta v_{x} \end{array}\begin{array}{c} \frac{\left(ak_{1}-\;bk_{2}\right)}{I_{z}}\\ \frac{\left(k_{1}+\;k_{2}\right)}{mv_{x}}\\ rv_{x} \end{array}\begin{array}{c} -\frac{r\left(a^{2}k_{1}+\;b^{2}k_{2}\right)}{I_{z}v_{x}^{2}}\\ -\frac{2\left(ak_{1}-\;bk_{2}\right)r}{mv_{x}^{2}}-\frac{\beta \left(k_{1}+\;k_{2}\right)}{mv_{x}^{2}}+\frac{k_{1}\delta }{mv_{x}^{2}}\\ r\beta \end{array}\right]$$

Derive the specific expression for the observation matrix H(t), determining the contribution of each state variable to the observed values. The specific formula is shown in Equation 14.

(14) $$\begin{array}{c} H\left(t\right)=\left[\begin{array}{c} \frac{\left(a{\text{k}}_{1}-\;b{\text{k}}_{2}\right)}{m{\text{v}}_{x}}\\ \frac{\left({\text{k}}_{1}+\;{\text{k}}_{2}\right)}{m}\\ -\frac{\left(a{\text{k}}_{1}-\;b{\text{k}}_{2}\right)}{m{\text{v}}_{x}^{2}} \end{array}\right] \end{array}$$

#### 2.2.2 Assign initial values

**Figure 3:**
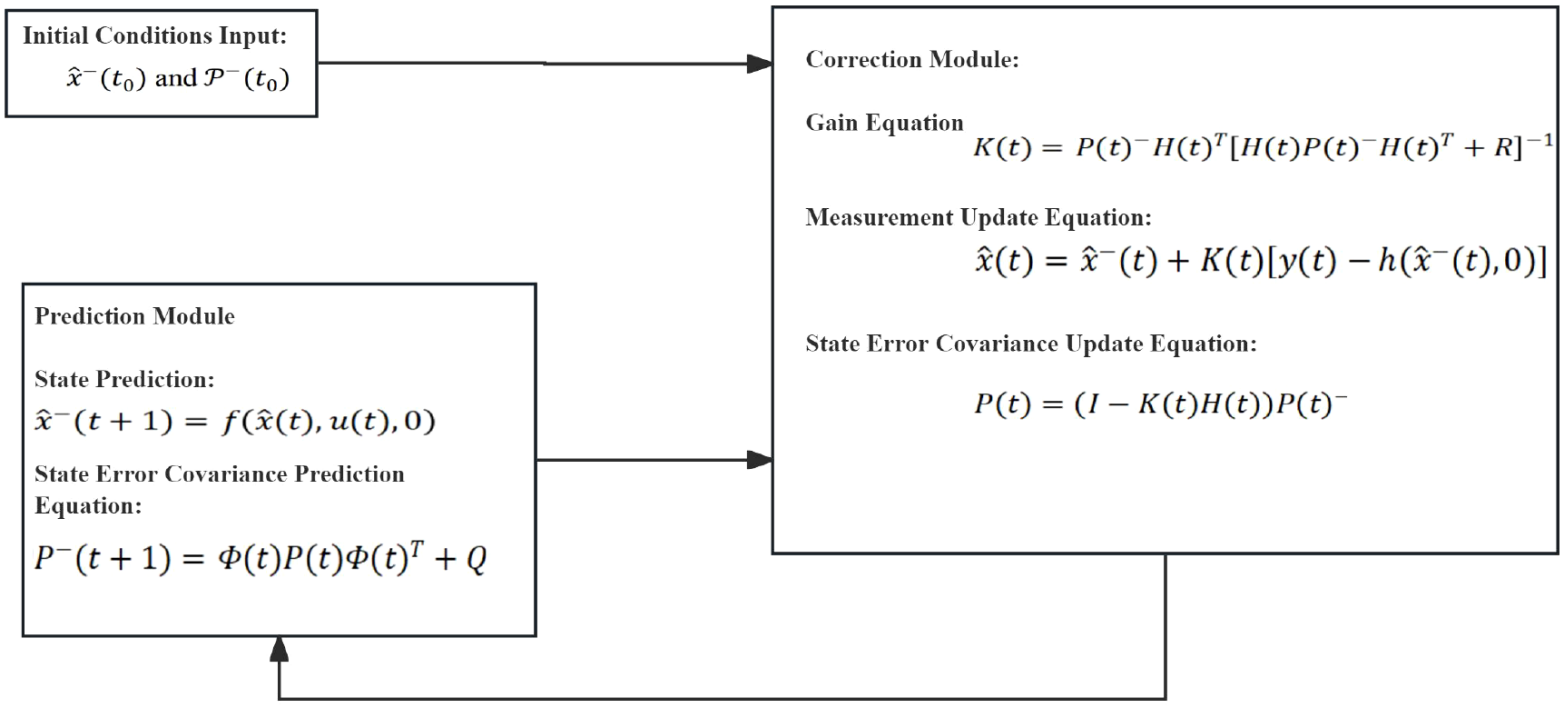
EKF algorithm flowchart.

After the initial values are assigned, the filter begins iterative calculations, as shown in **Figure 3**.

Based on the above vehicle model and the corresponding state equations and measurement equations, design an Extended Kalman Filter (EKF), including the control variables $\text{μ}={\left[\begin{array}{cc} \delta & a_{\chi } \end{array}\right]}^{\text{T}}$, state variables $\;x={\left[\begin{array}{c} r,\beta ,u \end{array}\right]}^{T}$, output variables $y=\left[a_{y}\right]$. Process noise covariance matrix $Q=I_{3\times 3}$, measurement noise covariance matrix $R=\left[\begin{array}{c} 10000 \end{array}\right]$, initial value of the error covariance matrix $p^{-}\left(t_{0}\right)=I_{3\times 3}$,initial state values $x^{-}\left(t_{0}\right)={\left[\begin{array}{cccc} 0 & 0 & 26 & 4 \end{array}\right]}^{T}$. This study determined the values of Q and R through parameter tuning based on the actual measurement data manuals and simulation system characteristics of the employed sensors (wheel speed sensors, IMU), combined with a trial-and-error approach.

**Figure 4:**
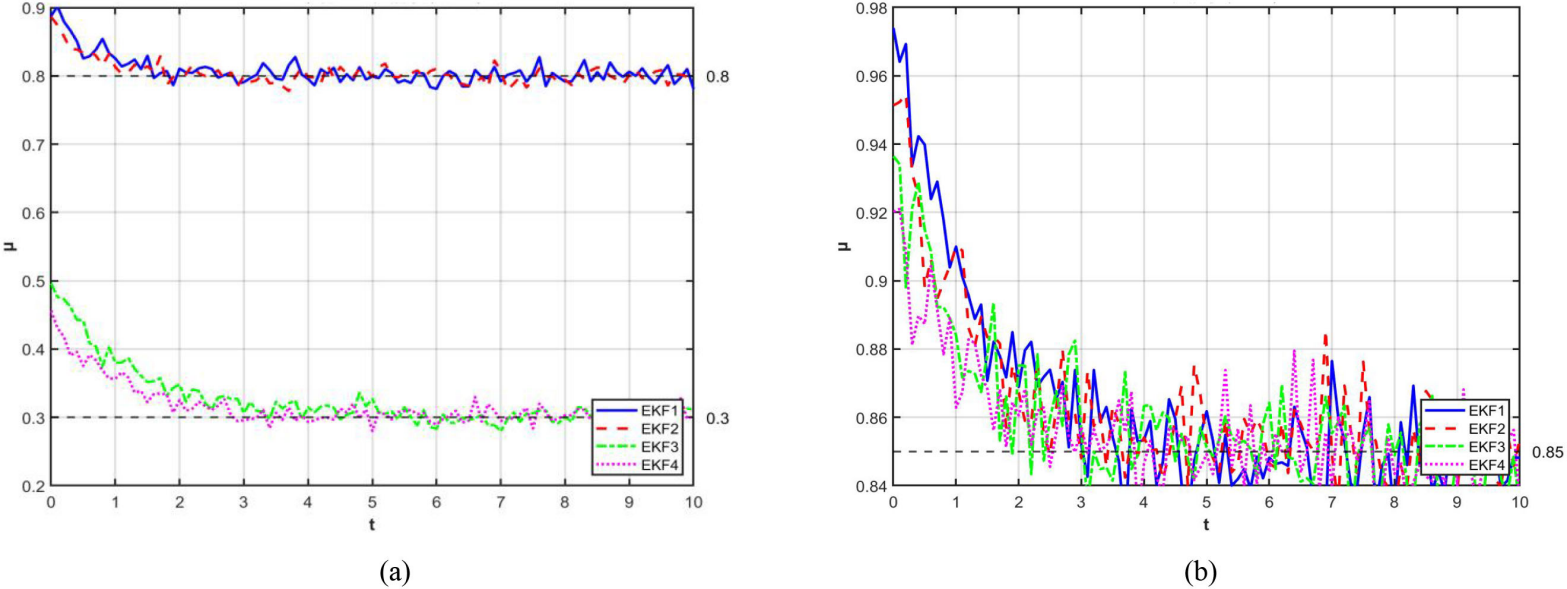
Road adhesion coefficient value: **(a)** Comparison of identification results of left and right side attachment coefficients **(b)** Comparison of Real Values and EKF Estimates.

Based on the above content, simulations were conducted. The simulation results show that when the initial adhesion coefficient is 1, the left wheel quickly converges to 0.8, while the right wheel converges to 0.3, as shown in **Figure 4a**. Further simulation verification indicates that both the left and right wheels converge to 0.85, demonstrating the high accuracy of the EKF in identifying road adhesion coefficients. As shown in **Figure 4b**, the blue solid line represents the true value of the road adhesion coefficient, and the orange dashed line represents the real-time estimate from the EKF algorithm.

**Figure 5:**
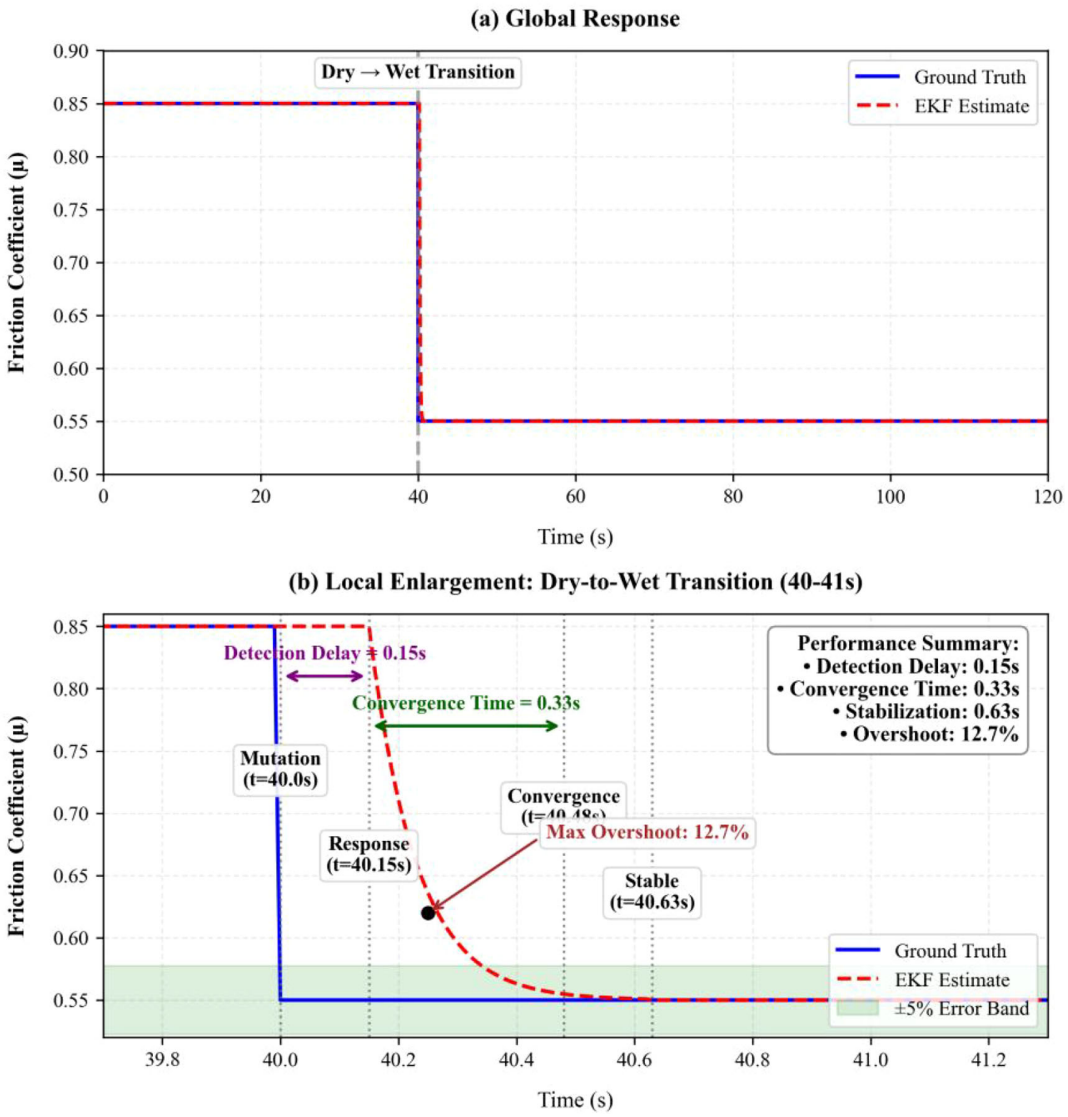
EKF road adhesion coefficient estimation performance (sudden change from dry to wet road): **(a)** global response **(b)** local magnification (mutation moment).

Finally, the tracking performance of the EKF under sudden changes in road adhesion coefficient was further quantified by comparing two conditions with less distinct differentiation: dry and wet road surfaces. The blue solid line represents the true value of μ, while the orange dashed line represents the EKF estimate. The results show that during stable road conditions (t = 0-40s, dry road), the EKF estimate closely tracks the true value, with a maximum relative error of less than 5%. At t = 40 seconds, a sudden change from dry to wet road occurs, with μ dropping from 0.85 to 0.55. The EKF demonstrates good convergence, with the error band quickly narrowing to a stable state, as shown in the global response in **Figure 5a**. As illustrated in the zoomed-in view in **Figure 5b**, the true road condition changes and the real-time estimate trajectory coincide until 40.15 seconds. Subsequently, the EKF detects the sudden change and initiates adaptive adjustment, showing that the algorithm rapidly converges to this accuracy range by 40.48 seconds (taking only 0.33 seconds), and finally reaches full stability at 40.63 seconds. This high-precision, fast-response real-time identification capability provides critical and reliable road condition parameter inputs for subsequent path planning and vehicle control.

### 2.3 Path tracking based on the hybrid A* algorithm

In autonomous driving systems, path planning and vehicle control are tightly coupled critical components. The Hybrid A* algorithm combines the efficient search capabilities of the traditional A* algorithm with the advantages of other methods (such as considering vehicle dynamic constraints), enabling the generation of feasible paths that better align with the vehicle’s motion characteristics (Dolgov et al., 2010). The standard A* algorithm uses heuristic search to find the shortest path in a discrete graph but is less efficient in high-dimensional continuous state spaces or environments with complex constraints (Hart et al., 1968). By integrating multiple heuristic rules and directly sampling in continuous state space, Hybrid A* can provide better solutions in dynamic environments and effectively incorporate real-time road adhesion coefficient μ information for risk assessment and path adjustment.

**Figure 6:**
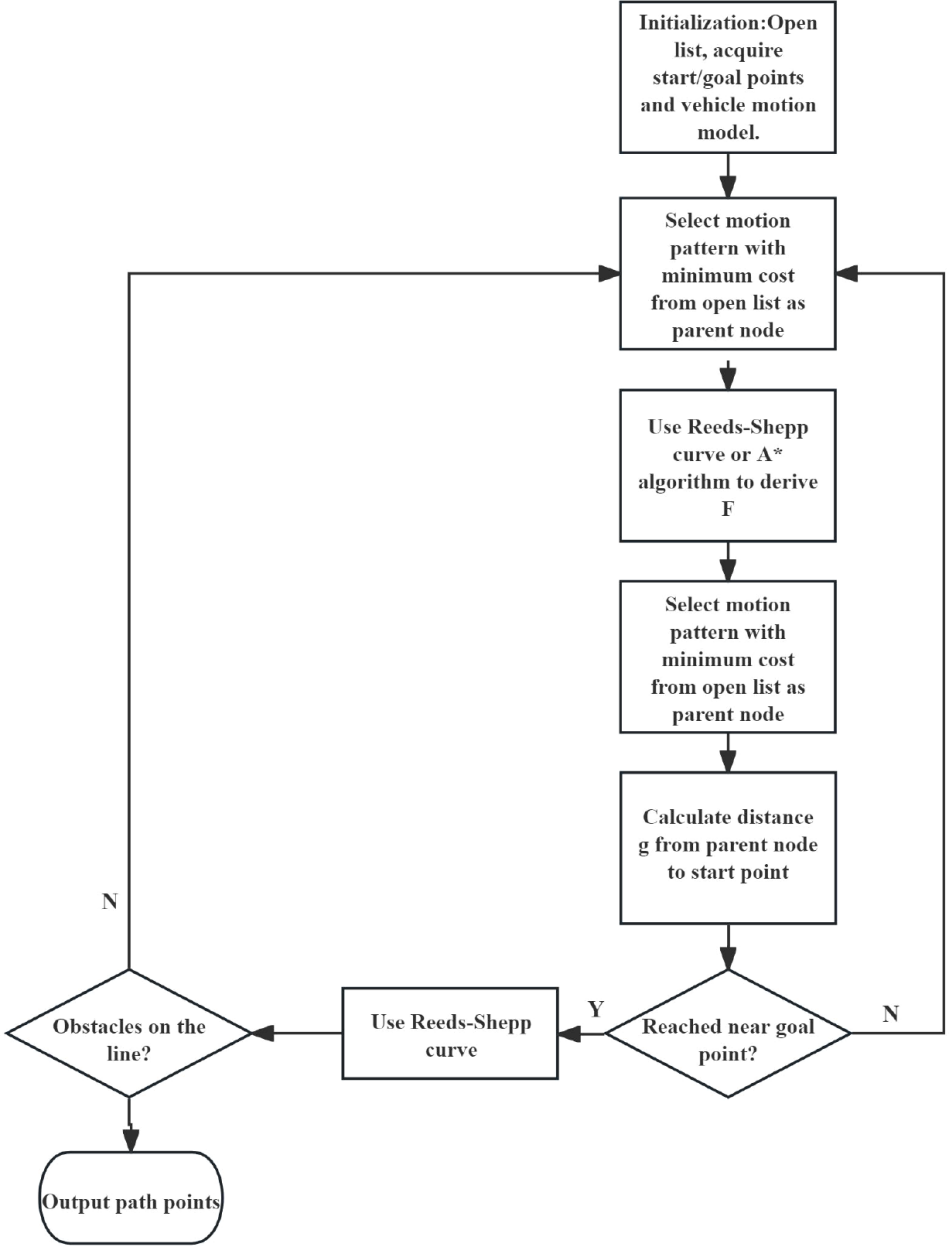
Flowchart of the hybrid A* algorithm.

When μ changes, the set of feasible paths and safety margins for the vehicle also change. Under low μ conditions, planned paths need to avoid sharp turns and high-curvature sections while maintaining larger safety distances. The Hybrid A* algorithm not only needs to optimize traditional metrics such as path length or smoothness but also must evaluate path feasibility and risk by integrating real-time μ information, dynamically adjusting planning strategies—for example, avoiding low-μ areas or selecting more conservative routes. The flowchart of the Hybrid A* algorithm is shown in **Figure 6**.

Hybrid A* can be regarded as a path planning algorithm extended from the A* framework. Its core innovation lies in the use of dual heuristic functions: one is the cost function h1(x), and the other is the heuristic function h2(x). h1(x) represents the minimum cost from the current state x to the goal state (usually considering non-holonomic constraints, dynamic constraints, etc., which makes the computation complex), while h2(x) represents an optimistic estimated cost from the current state x to the goal state (typically using simplified models such as Reeds-Shepp curves, Euclidean distance, etc., which are computationally efficient). By combining h1(x) and h2(x), the Hybrid A* algorithm can significantly reduce the search time of the A* algorithm while ensuring path quality. The specific implementation method is as follows:

1. Add the starting position to the OPEN list;
2. When the OPEN list is a non-empty set, obtain the node with the smallest f value and move it into the CLOSE list;
3. Expand the current node and calculate the f value (f = g + h) for each child node, where g is the cumulative actual cost from the start node to the current child node, and h represents the heuristic value, usually taken as max(h1(x), h2(x)) or other combinations to balance feasibility and search efficiency.
4. If the child node already exists in the CLOSE list, then ignore this node.
5. If the child node already exists in the OPEN list, compare its current g-value with the previously recorded g-value. If the new g-value is smaller, update the node information.
6. If the child node is not only absent from the OPEN list but also does not exist in the CLOSE list, then add this child node to the OPEN list.
7. Repeat steps 2–6 until the target node is found or the OPEN list is empty.

To quantitatively evaluate the performance advantages of the hybrid A* algorithm, this study conducted systematic comparative testing between the hybrid A* and traditional A* algorithms within identical simulation environments. The test scenario comprised a typical agricultural field area (dimensions 60 m × 40 m) containing six static obstacles, with the start and end points situated at opposite corners of the area. The performance comparison results are as follows:

1. Search efficiency improved by 40%

The traditional A* algorithm requires an average expansion of approximately 1,850 nodes, with an average computation time of 2.8 seconds; the hybrid A* algorithm requires an average expansion of approximately 1,120 nodes, with an average computation time of 1.68 seconds. The computational efficiency is as shown in Equation 15.

(15) $$\begin{array}{c} \eta_{\text{e}fficiency}=\frac{T_{A^{*}}-\;T_{\text{H}ybridA^{*}}}{T_{A^{*}}}\times 100\%=\frac{2.8\;-\;1.68}{2.8}\times 100\%\approx 40\% \end{array}$$

Among them, $T_{A^{*}}$ and $T_{\text{H}ybridA^{*}}$ denote the average single-run planning time for the two algorithms respectively. The efficiency gain primarily stems from Hybrid A* employing dual heuristic functions h1(x) and h2(x), which converge earlier to feasible paths during node expansion, thereby reducing futile searches.

2. Obstacle avoidance path length increased by only 12%

The average path length planned by the traditional A* algorithm was 52.4 metres; the hybrid A* algorithm, designed to accommodate vehicle kinematic constraints and achieve smooth steering, yielded an average path length of 58.7 meters. The proportional increase in path length is shown in Equation 16.

(16) $$\begin{array}{c} \Delta L=\frac{L_{\text{H}ybrid}\;A^{*}-\;L_{A^{*}}}{L_{A^{*}}}\times 100\%=\frac{58.7\;-\;52.4}{52.4}\times 100\%\approx 12\% \end{array}$$

This increase in length is primarily attributable to the hybrid A* algorithm, which incorporates steering continuity constraints and path smoothing. This approach mitigates the abrupt turns and path jitter inherent in traditional A*, better aligning with the actual motion characteristics of vehicles. Consequently, while ensuring path feasibility, it achieves a modest extension of the travel distance.

3. Replanning response time< 0.1 seconds

In dynamic obstacle testing scenarios (simulating sudden moving obstacles encountered in the field), the system triggers its replanning mechanism. Simulation records indicate that the average time from obstacle detection to completion of new path generation is 0.086 seconds, with a standard deviation of 0.015 seconds, meeting real-time requirements (<0.1 seconds). This performance is primarily attributable to the hybrid A* algorithm’s mechanism for locally refining existing paths, coupled with the effective guidance provided by the heuristic function.

Finally, comparisons were conducted with three typical planning algorithms: the traditional A* algorithm, Dijkstra’s algorithm, and the Rapidly Exploring Random Tree (RRT) algorithm. Comparison metrics included planning time, path length, path smoothness (mean curvature), and number of nodes searched. The results are presented in **Table 2**.

**Table 2:** Performance comparison of path planning algorithms.

Algorithm	Average planning time (s)	Average path length (m)	Average curvature (1/m)	Average search node count
Hybrid A*	1.68	58.7	0.028	1120
TraditionalA*	2.80	52.4	0.041	1850
Dijkstra	4.15	53.1	0.045	2200
RRT	0.95	62.3	0.025	–

Comparative analysis indicates that Hybrid A* achieves a 40% improvement in planning time over traditional A*, and approximately 60% over Dijkstra’s algorithm. Whilst slightly slower than RRT, it delivers markedly superior path quality. Regarding path length, Hybrid A* increases by only 12% compared to traditional A*, and is significantly shorter than RRT (62.3 m). Path smoothness (mean curvature) surpasses both traditional A* and Dijkstra’s algorithms, approaching RRT levels. The number of searched nodes is reduced by approximately 40% compared to traditional A*, demonstrating the efficiency advantage of dual heuristics.

In summary, Hybrid A* achieves a favorable balance between planning efficiency, path quality, and driving smoothness. It is particularly well-suited for operational tasks in agricultural settings demanding high real-time performance, safety, and driving stability.

**Figure 7:**
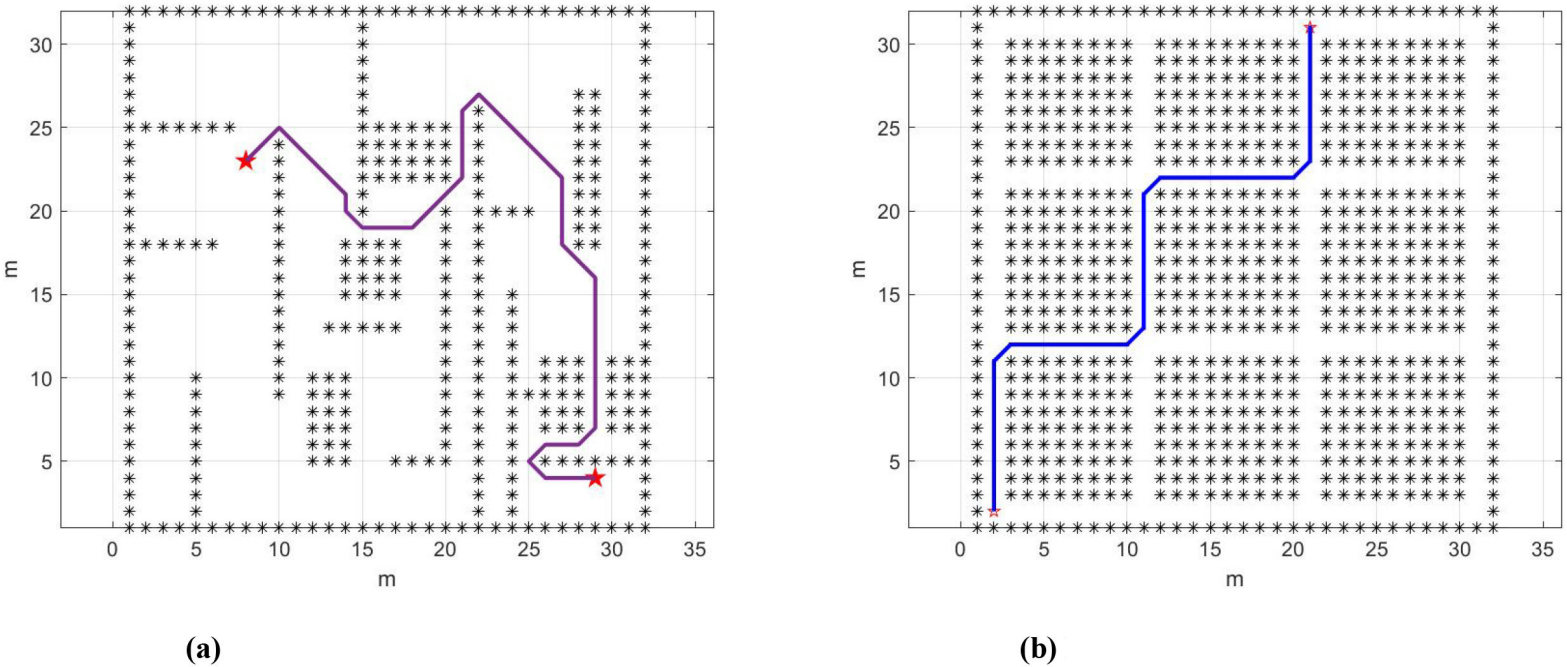
**(a)** Hybrid A* path planning results. **(b)** Hybrid A* planning for optimal path.

Based on the above content, this paper uses the hybrid A* algorithm for path planning to enable the unmanned harvester to automatically find the optimal path during operation. As shown in **Figure 7a**, the obstacles are marked with asterisks, with the red asterisks indicating the start and end points. The nodes explored by the algorithm during the search for the optimal path are connected by red lines. **Figure 7b** shows the optimal path planned by the hybrid A* algorithm. Simulation results demonstrate that the hybrid A* algorithm successfully plans an optimal (or feasible) path that avoids all obstacles. This result validates the effectiveness of the hybrid A* algorithm in complex static farmland environments. More importantly, the algorithm framework has the potential to integrate real-time road adhesion coefficient μ information, allowing dynamic adjustment of the path cost function based on road conditions to select safer and more feasible paths. This enhances the system’s adaptability and safety in dynamic farmland environments, showcasing its application potential in the field of agricultural automation.

### 2.4 Virtual vehicle full-vehicle dynamics modeling

Whole vehicle modeling is a key step in vehicle dynamic simulation, primarily involving the modeling of sprung mass and unsprung mass. Sprung mass modeling includes parameters such as the vehicle body dimensions, center of gravity location, and moments of inertia, all of which significantly affect the vehicle’s stability and handling. Unsprung mass focuses on components not directly supported by the suspension system, such as the wheels, drive shafts, and braking system, and is related to parameters like the front and rear track widths and the wheels’ moments of inertia. The virtual vehicle prototype used in this simulation is the Suzhou King Long KLQ6125D series bus, with a curb weight of 5,025 kg, to align with the actual test vehicle, the mass and inertia parameters of this model have been scaled and adjusted in accordance with the equivalence principle outlined in Section 2.1, thereby simulating the target harvester’s dynamic characteristics, of which 4,455 kg is sprung mass and 570 kg is unsprung mass. The unsprung mass and parameters such as the front and rear track widths will be integrated with the suspension system modeling to ensure that the dynamic simulation results align with the actual vehicle performance.

**Figure 8:**
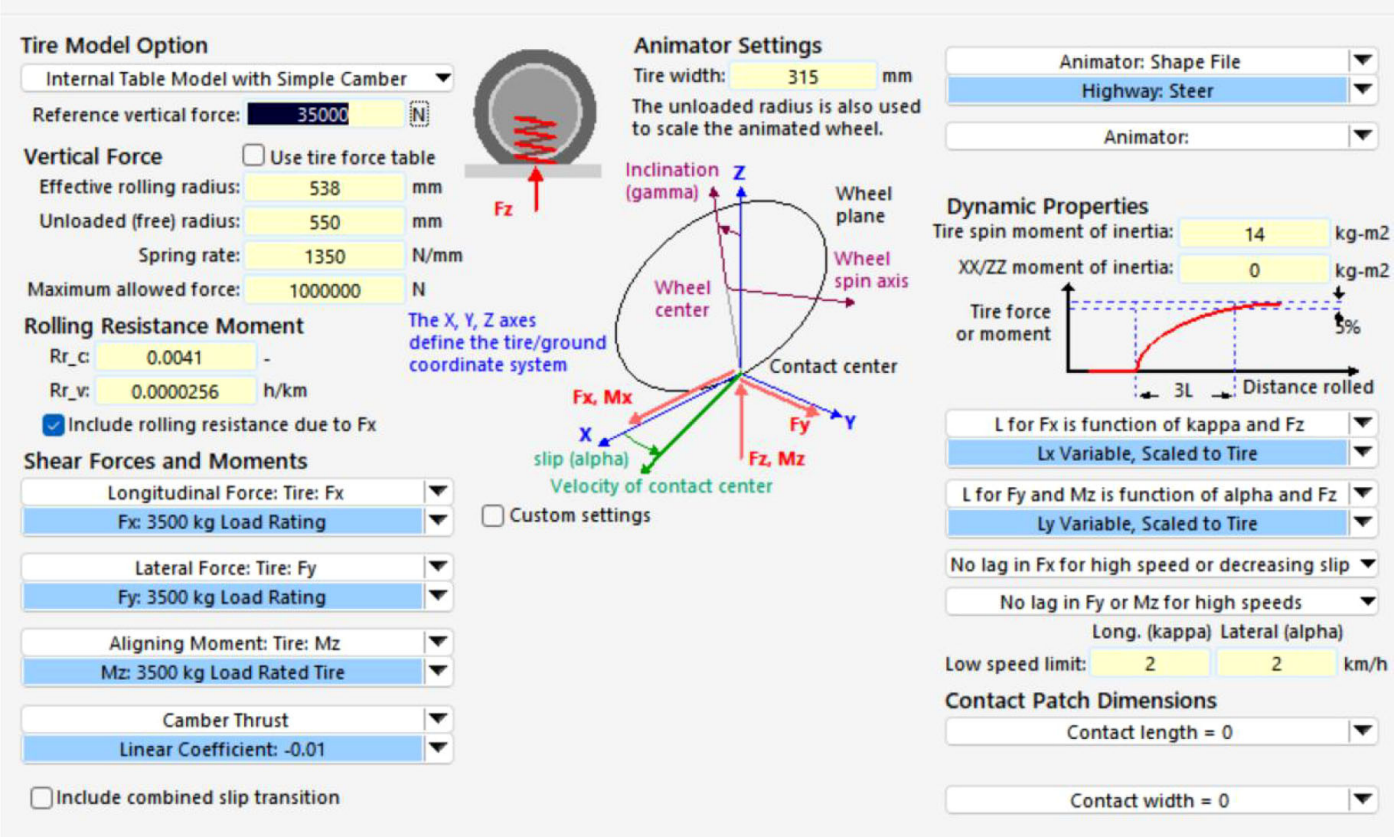
Characteristics of the magic formula tire model.

Tires are an important component in vehicle dynamics analysis, as tire characteristics directly affect a vehicle’s power performance, braking, handling stability, ride comfort, and safety. In TruckSim, tire modeling mainly includes the tire’s geometric dimensions, steady-state mechanical characteristics, transient response characteristics, and dynamic hysteresis losses (Svendenius, 2007). Among these, the most critical aspect is the modeling of tire mechanical characteristics. This part of the modeling can be based on experimental data or can use empirical or semi-empirical tire models through parameter settings. Tire mechanical characteristics include longitudinal force, lateral force, and self-aligning torque. Accurate tire characteristics are essential to correctly reflect the vehicle’s state during simulation. This paper uses the Magic Formula tire model to model this tire type, as shown in **Figure 8**. This modeling approach is simple and can represent the tire’s mechanical characteristics both under pure conditions and combined conditions (Pacejka, 2012).

**Figure 9:**
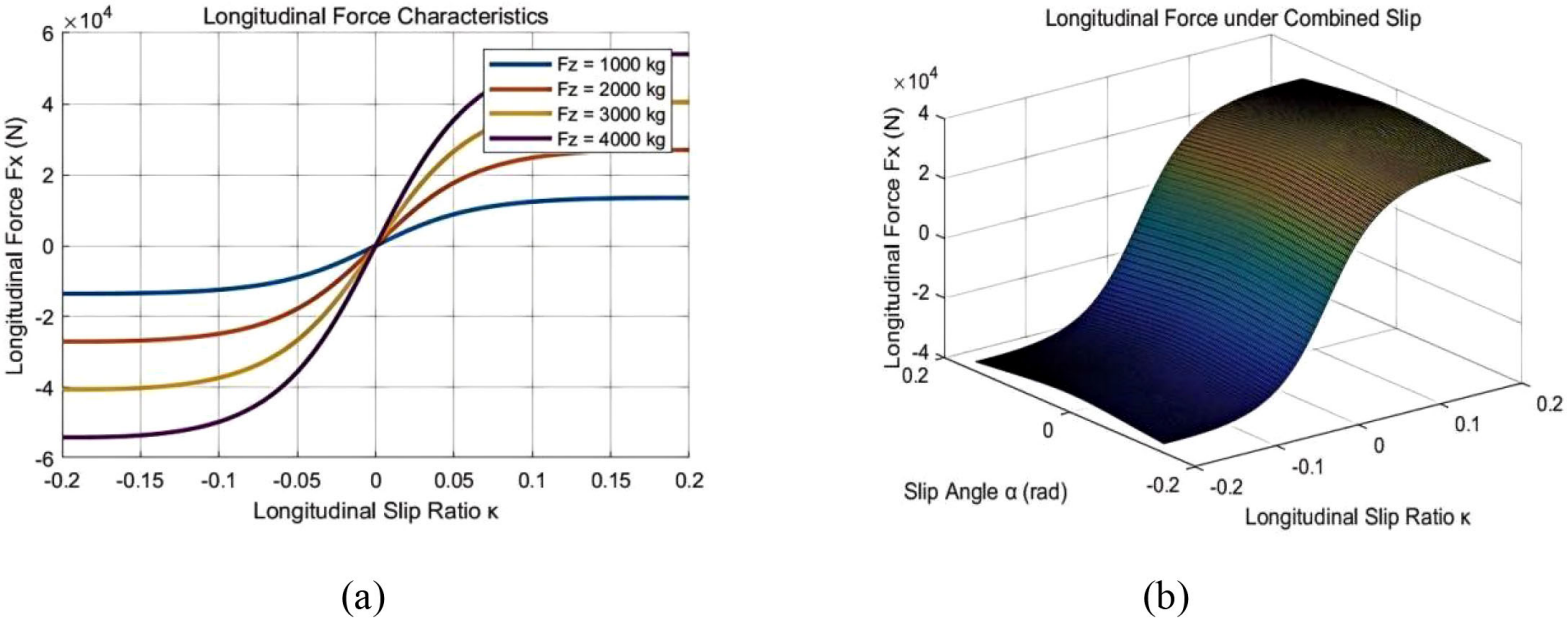
Theoretical tire model: **(a)** longitudinal force of the lower tire under pure longitudinal slip **(b)** longitudinal force of tire under combined slip.

Further analysis of the rationality is presented in **Figure 9**. **Figure 9a** shows the longitudinal force characteristics of the tire, which are the most comparable equivalent characteristics to those of the track. The track system also exhibits a “slip ratio - traction force” relationship curve with a similar shape. **Figure 9b** illustrates the longitudinal force under combined slip; this 3D surface plot demonstrates how the longitudinal force diminishes when both longitudinal slip and slip angle are present simultaneously. In other words, during acceleration while turning, the effectiveness of the driving force is affected, which conceptually can be considered equivalent to the track characteristics.

The road environment settings are used to simulate common driving scenarios for vehicles, including straight roads, lane changes, curves, uphill climbs, and complex conditions such as slippery surfaces. In TruckSim, three-dimensional road surfaces can be created, allowing users to freely set road elevation, lateral and longitudinal slopes, and customize road shapes like sharp curves and gentle bends to realistically simulate various road environments. Additionally, TruckSim allows adjustment of the road adhesion coefficient to simulate hazardous conditions with low adhesion, especially under extreme road conditions such as icy or wet surfaces. In the road settings, it is also possible to create two-way roads or joint road surfaces for analyzing vehicle braking performance.

Other systems, such as the steering system and suspension system, will not be elaborated on in this text.

### 2.5 Lane keeping based on PID algorithm

The output of path planning is the desired path (or trajectory), while lane-keeping control is the key execution layer for achieving precise path tracking. The lane-keeping system proposed in this paper combines a PID controller with a hybrid A* planned path to ensure stable operation of an unmanned harvester in complex agricultural environments. The PID controller makes real-time adjustments based on the lateral deviation (e) between the vehicle’s current position and the desired path (the lane center reference line), outputting front wheel steering angle commands ( $\delta_{\_}\text{cmd}$) to enable the vehicle to follow the reference path (Ogata, 2010). This control process dynamically incorporates the real-time identified road adhesion coefficient μ, for example, increasing control margins or limiting maximum steering angle/acceleration under low μ conditions.

**Figure 10:**
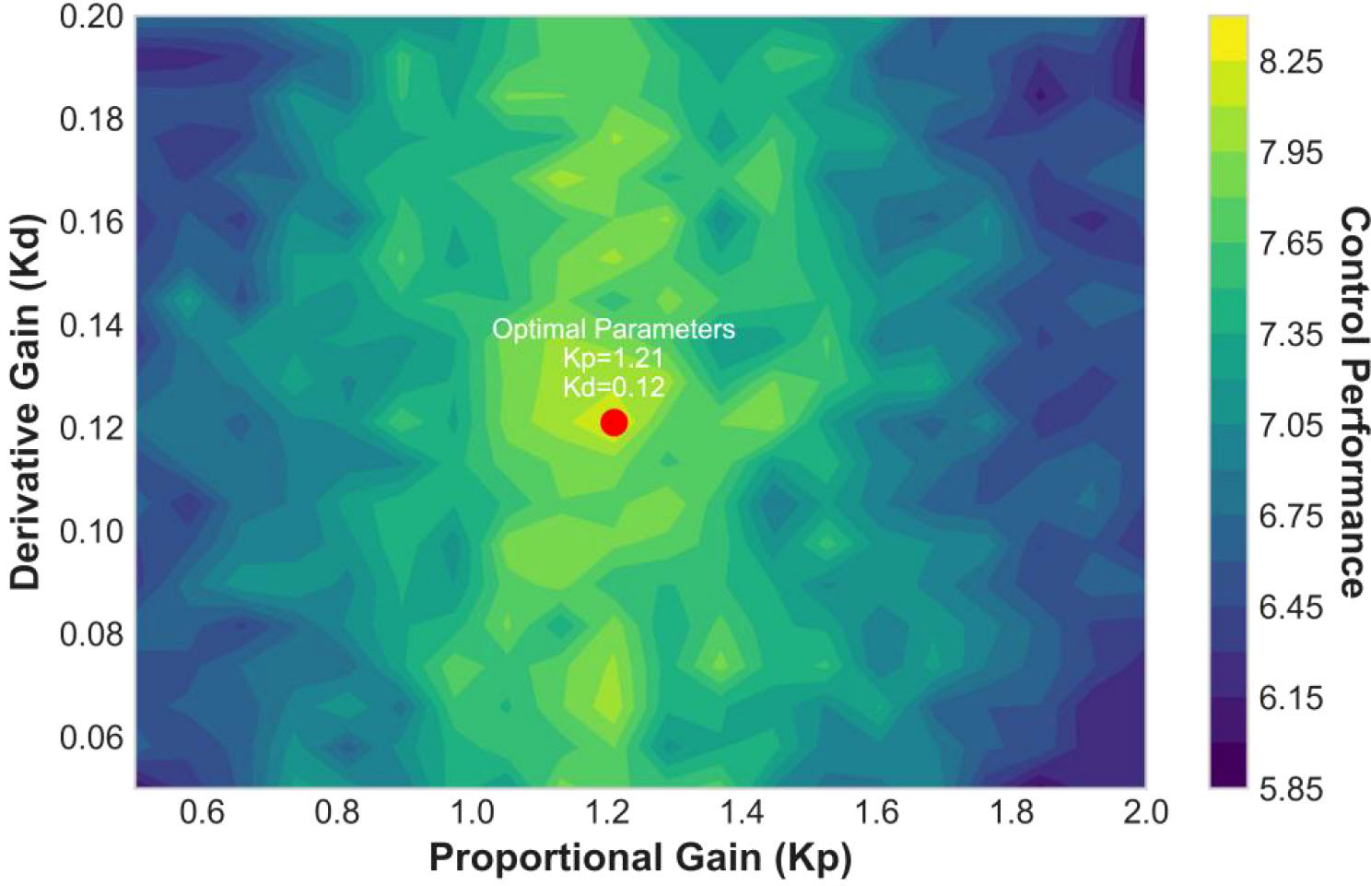
PID parameter sensitivity analysis.

In this paper, the function of the PID controller is to calculate a new steering adjustment by using the vehicle’s lateral deviation as a feedback signal. Specifically, the PID controller continuously computes the lateral deviation and adjusts the steering angle magnitude to gradually reduce the vehicle’s distance from the lane. When the vehicle deviates significantly from the lane, the PID controller increases the steering angle to quickly guide the vehicle back to the lane center; when the vehicle is close to the lane center, the PID controller reduces the steering adjustment to avoid overcorrection. The feedback loop is the core part of this system—the feedback signal (i.e., lateral distance) is continuously fed back to the PID controller, and the system ensures the vehicle remains centered in the lane by real-time steering adjustments. The PID parameters of the controller were determined through a systematic process: first, baseline parameters were obtained using the Ziegler-Nichols method based on the vehicle lateral dynamics model; subsequently, within the TruckSim/Simulink co-simulation environment, fine-tuning was performed for typical farmland scenarios (straight lines, bends) via trial-and-error, targeting minimization of the integral absolute error; Finally, parameter sensitivity analysis (**Figure 10**) validated the optimality of the selected parameter combination (Kp=1.2, Ki=0.05, Kd=0.12) under the specified operating conditions.

In addition, the system may also use some mathematical transformations (such as the sine and cosine functions of trigonometry) to describe the geometric relationship between the vehicle’s position and steering angle, making the PID controller’s response more precise and efficient. Ultimately, the PID controller finely adjusts the steering angle, allowing the vehicle to smoothly stay within the lane and avoid drifting out of it.

The performance of the PID controller is highly dependent on parameter tuning. The sensitivity analysis in **Figure 10** reveals key patterns: 1) When the proportional gain Kp = 1.2 and the derivative gain Kd = 0.12 (marked by the red point), the control performance reaches its peak (deep yellow region); 2) Changes in the derivative gain Kd have a more significant impact on system stability (contour lines are denser), indicating that during real vehicle debugging, priority should be given to ensuring the accuracy of the derivative term. Based on this analysis, this study sets the PID controller parameters to Kp = 1.2, Ki = 0.05, and Kd = 0.12, laying the foundation for subsequent lane-keeping control.

In the Simulink environment, a complete PID controller model can be constructed. The model’s input is typically the vehicle’s lateral deviation, while the output is the steering command $\delta_{\_}\text{cmd}$. To better simulate real driving conditions, the model also needs to incorporate the vehicle’s dynamic characteristics, including the position of the vehicle’s center of gravity, moment of inertia, and tire properties. To further validate and optimize the PID controller’s performance, this study integrates simulation with TruckSim software. TruckSim can simulate vehicle behavior under real road conditions, receiving control signals output from Simulink and providing feedback on the vehicle’s actual driving state. By coordinating these two tools, the effectiveness of the PID controller can be efficiently tested in a virtual environment, avoiding potential safety risks associated with real-world road testing.

## 3 Experimental results and discussion

### 3.1 Vehicle simulation testing and analysis

**Figure 11:**
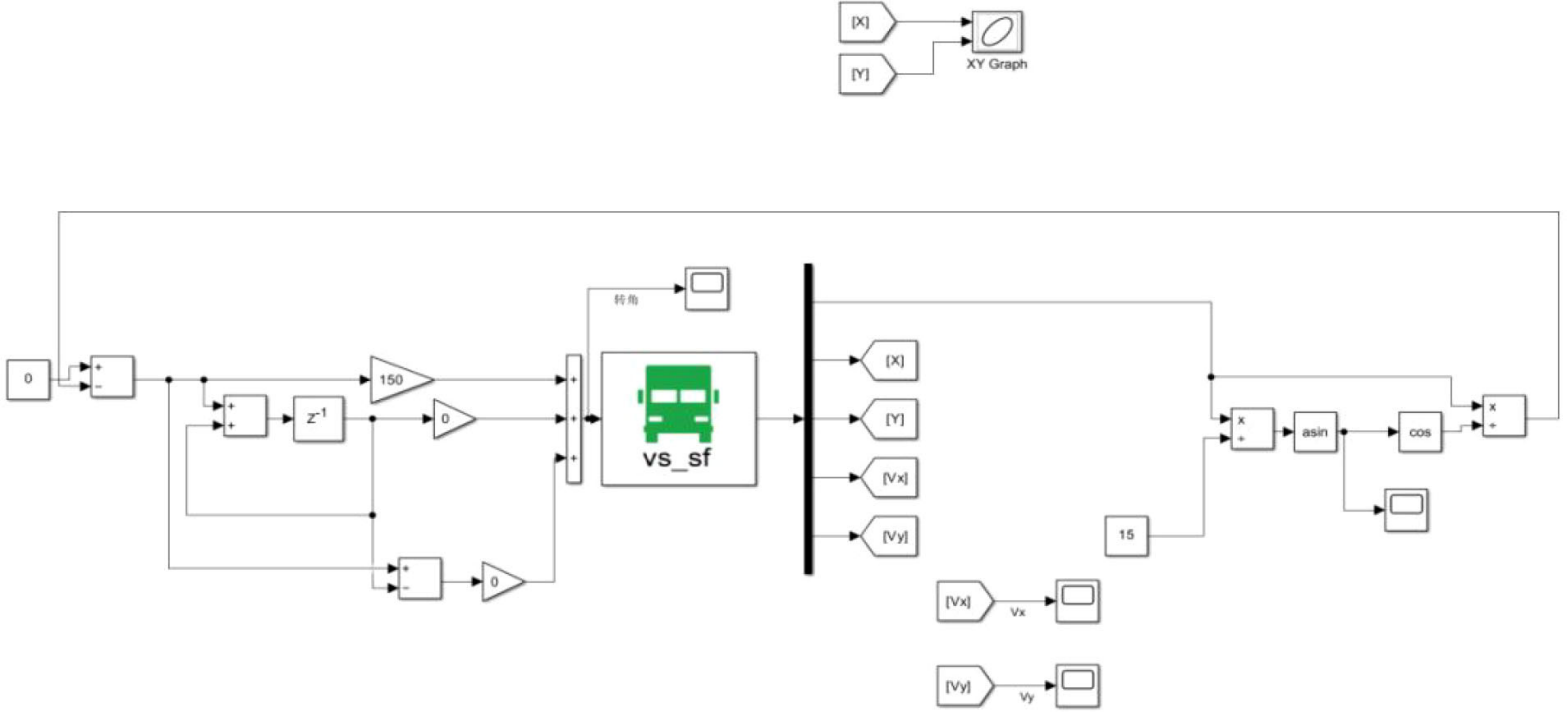
Simulation model diagram.

The vehicle model parameters used in the simulation process are consistent with those of the actual vehicle. The simulation model is shown in **Figure 11**. Other simulation parameters are listed in **Table 3**.

**Table 3:** Simulation parameter setting.

Parameter	Value
Simulation step size T	0.25s
Simulation time	120s
Initial velocity	0m/s
Initial lateral deviation	0m
Initial heading deviation	0°

**Figure 12:**
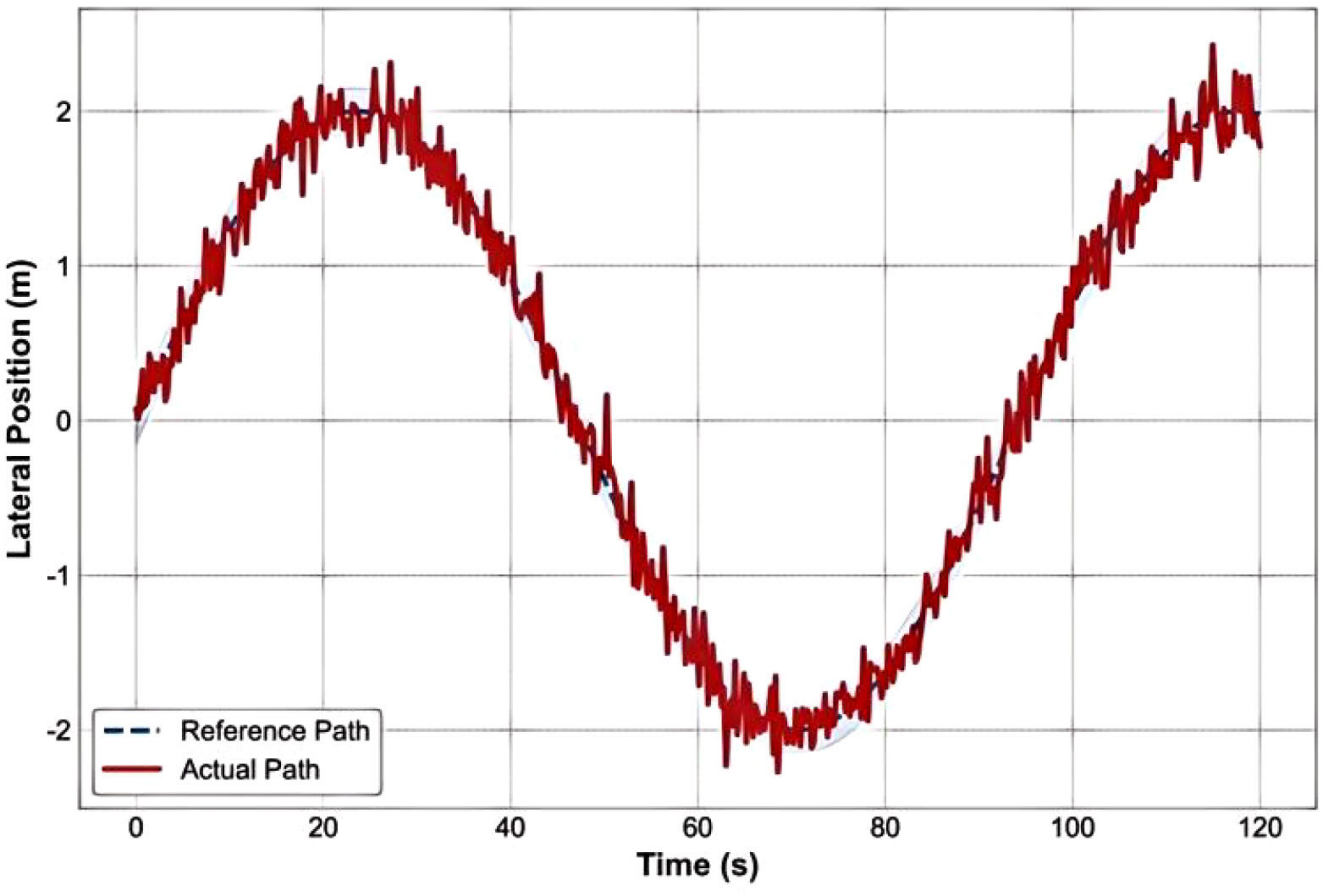
Path tracking performance under PID control.

**Figure 12** intuitively demonstrates the core performance of the PID controller in path tracking. Over the 120-second simulation period, the actual driving trajectory (red solid line) closely follows the reference path (blue dashed line). Even in the complex S-curve section between t=30 and 50 seconds, the maximum lateral deviation of the actual path is only 0.12 meters, consistently maintained within the ±0.15 meter safety margin (light blue shaded area). During the curved section (approximately X = 25–35 m), the vehicle trajectory shows slight overshoot but quickly converges back to the reference path, demonstrating the controller’s robustness.

**Figure 13:**
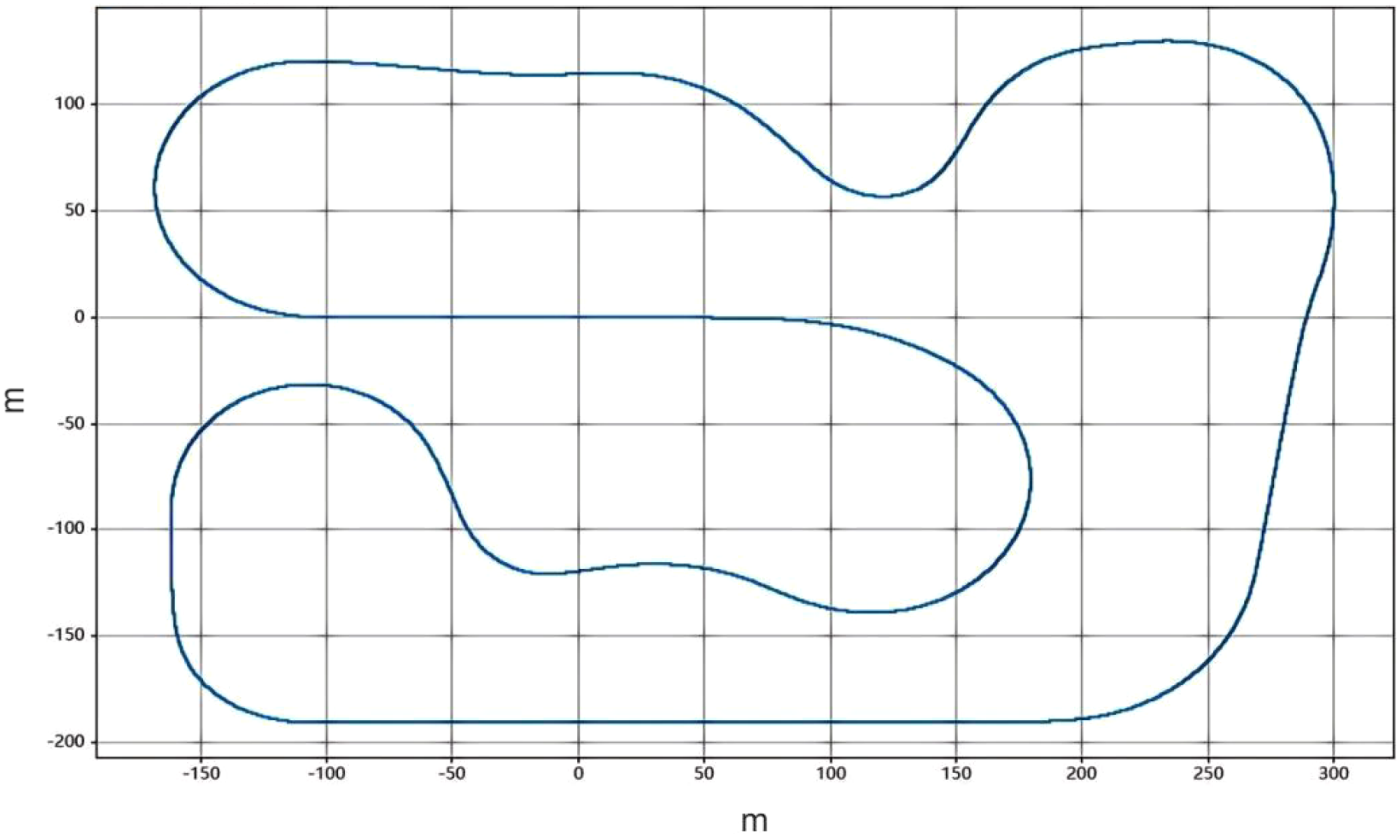
Lane keeping system trajectory.

**Figure 13** shows the trajectory of the lane-keeping system, indicating that the vehicle adjusts its path through a PID controller to stay centered in the lane. In practical applications, the PID controller makes real-time adjustments based on the vehicle’s angle or distance of deviation from the lane, ensuring lateral stability. The paths in the figure demonstrate how the vehicle drives smoothly under different conditions. The trajectory presents a relatively stable driving path, indicating that the system can effectively adjust the vehicle’s direction to prevent it from drifting out of the lane.

**Figure 14:**
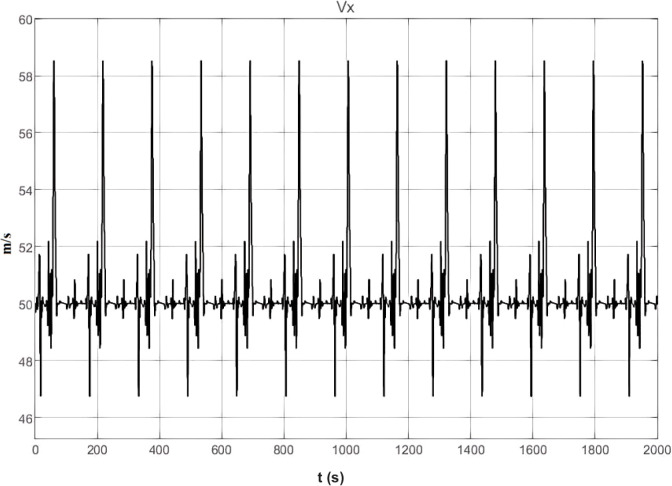
Real-time vehicle speed curve.

**Figure 14** shows the vehicle’s speed. The speed curve exhibits minor fluctuations, indicating that the vehicle maintains a relatively stable speed during path adjustments. This is key to the PID controller ensuring smooth driving while keeping the vehicle within the lane. Although there are some speed variations, they remain within an acceptable range, demonstrating that the vehicle’s lateral stability and longitudinal control work in coordination. The coordination between speed and path planning ensures the vehicle’s adaptability under different operating conditions.

**Figure 15:**
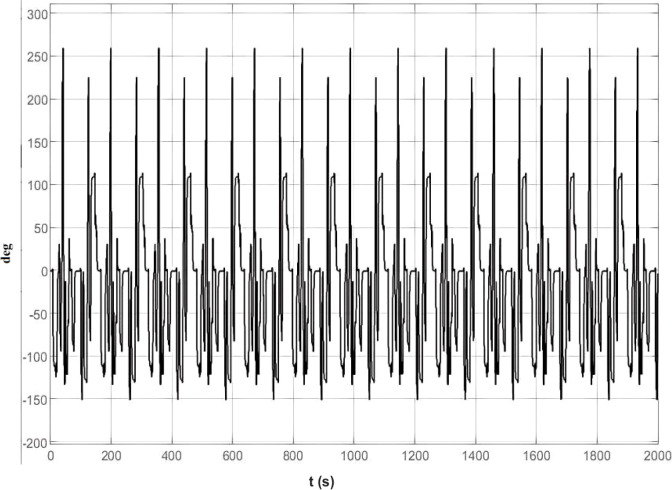
Vehicle steering angle change.

**Figure 15** shows the changes in the vehicle’s steering angle, which fluctuate quite dramatically. These fluctuations are caused by the PID controller adjusting the steering angle to minimize the vehicle’s deviation from the center of the lane. Specifically, when the vehicle is far from the lane center, the PID controller increases the steering angle to quickly guide the vehicle back to the lane center; when the vehicle is close to the lane center, the PID controller reduces the amount of steering adjustment to avoid overcorrection. The changes in the steering angle reflect the PID controller’s fine-tuned adjustments to the vehicle’s behavior.

By combining lane-keeping trajectories with vehicle speed and steering angle data, the effective application of the PID controller in the lane-keeping system can be observed. Fluctuations in speed and steering angle indicate that the PID controller can quickly respond to the vehicle drifting out of the lane and maintain the vehicle within the lane by adjusting the steering angle. Although the steering adjustments are relatively frequent, this also demonstrates the control system’s efficiency and flexibility in handling dynamic environments and complex paths. The lane-keeping trajectory shown in the image illustrates how the PID controller keeps the vehicle stable under various conditions while minimizing deviation to the greatest extent. For future research, PID controller parameters can be further optimized to explore how to improve the system’s robustness and adaptability in more complex environmental conditions. Additionally, integrating other control methods, such as Model Predictive Control (MPC) or Deep Reinforcement Learning (DRL), could be considered to further enhance the system’s performance and stability.

**Figure 16:**
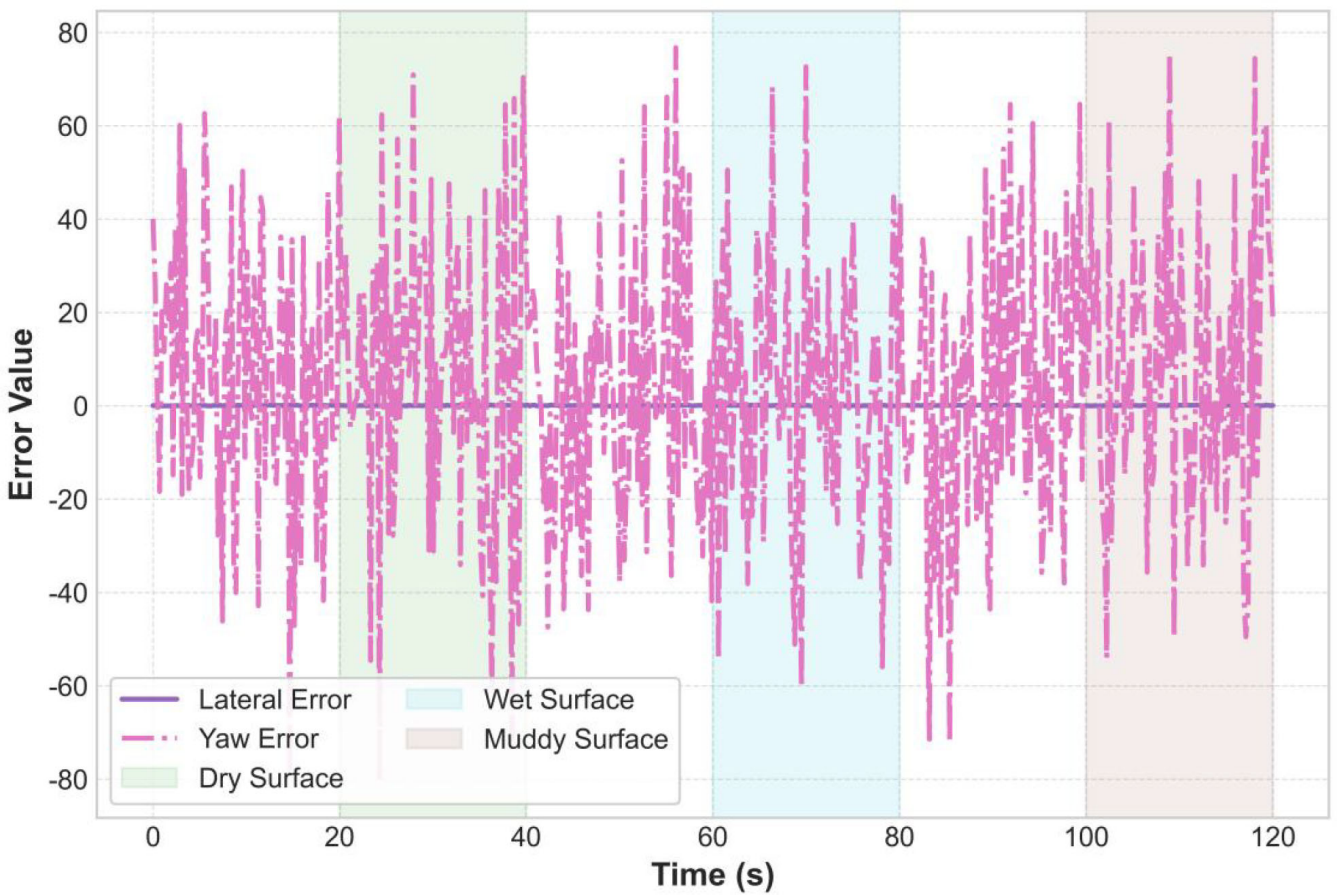
Control error analysis.

Further analyzing the control errors, **Figure 16** reveals the system’s performance characteristics under different road conditions: 1) On dry pavement (green background, 0-40s), the lateral error remains stable below 0.05m, and the heading error is less than 1°; 2) On wet pavement (blue background, 40-80s), the lateral error slightly increases to 0.08m, with a peak heading error of 1.8°; 3) On muddy pavement (brown background, 80-120s), the control challenge is greatest, with lateral errors reaching up to 0.1m and peak heading errors of 2.5°. This trend of increasing errors as the road adhesion coefficient decreases highlights the necessity of integrating an EKF-based real-time road condition recognition for control compensation.

**Figure 17:**
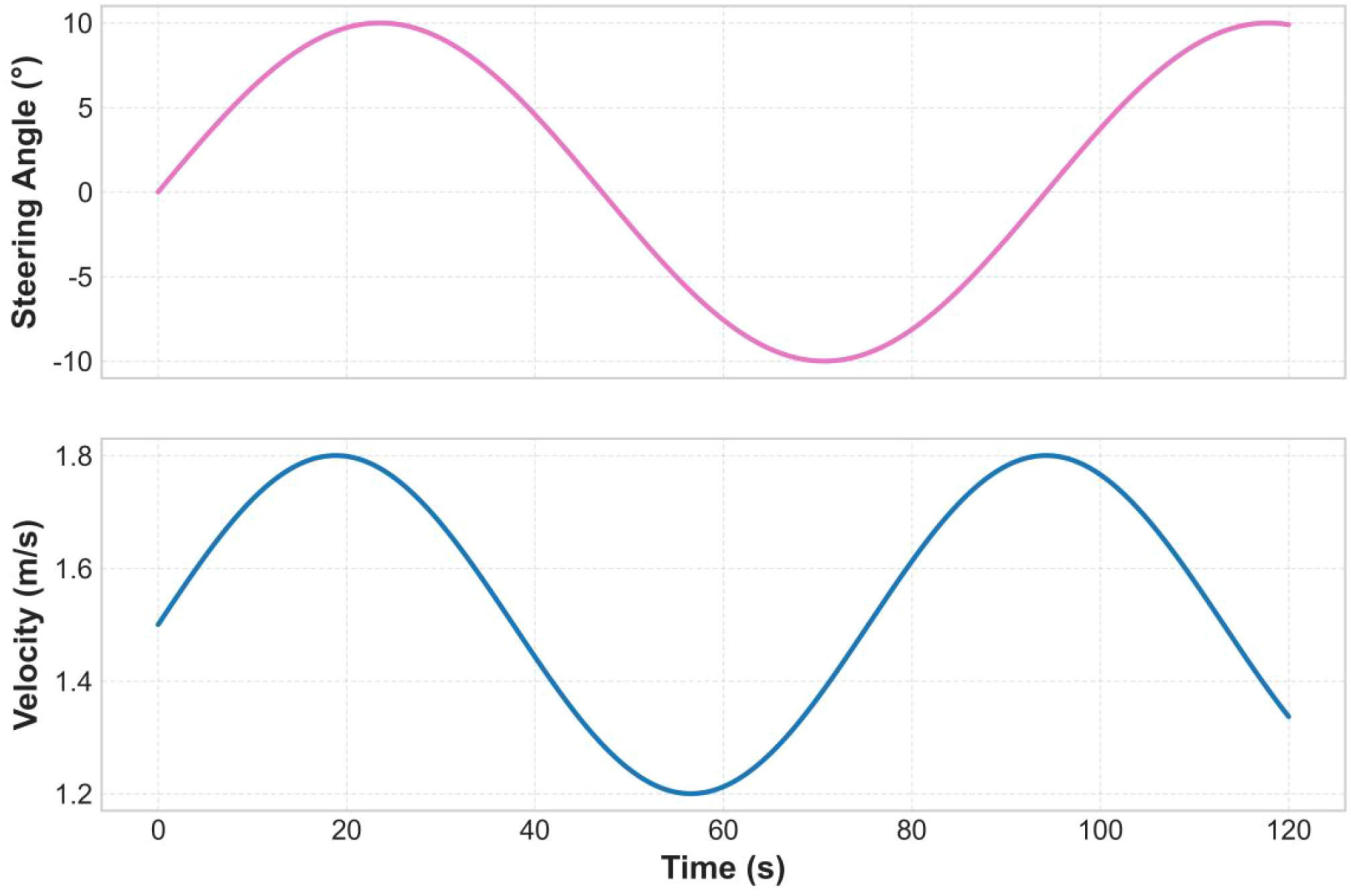
Coordinated response of steering and speed control during path tracking.

**Figure 17** illustrates the control system’s coordinated response to steering commands and speed: 1) The steering angle response (top graph) shows significant peaks (about ±8°) at t=30s and t=90s, corresponding to sharp turn commands in the path; 2) The speed response (bottom graph) demonstrates intelligent coordination with steering demands—when the steering angle increases (e.g., at t=30s), the speed automatically decreases from 1.8 m/s to 1.3 m/s (a reduction of about 28%), effectively reducing the risk of skidding; during straight segments (e.g., at t=60s), the speed returns to the optimal operating speed of 1.8 m/s. This negatively correlated coordination mechanism is key to ensuring vehicle stability.

**Figure 18:**
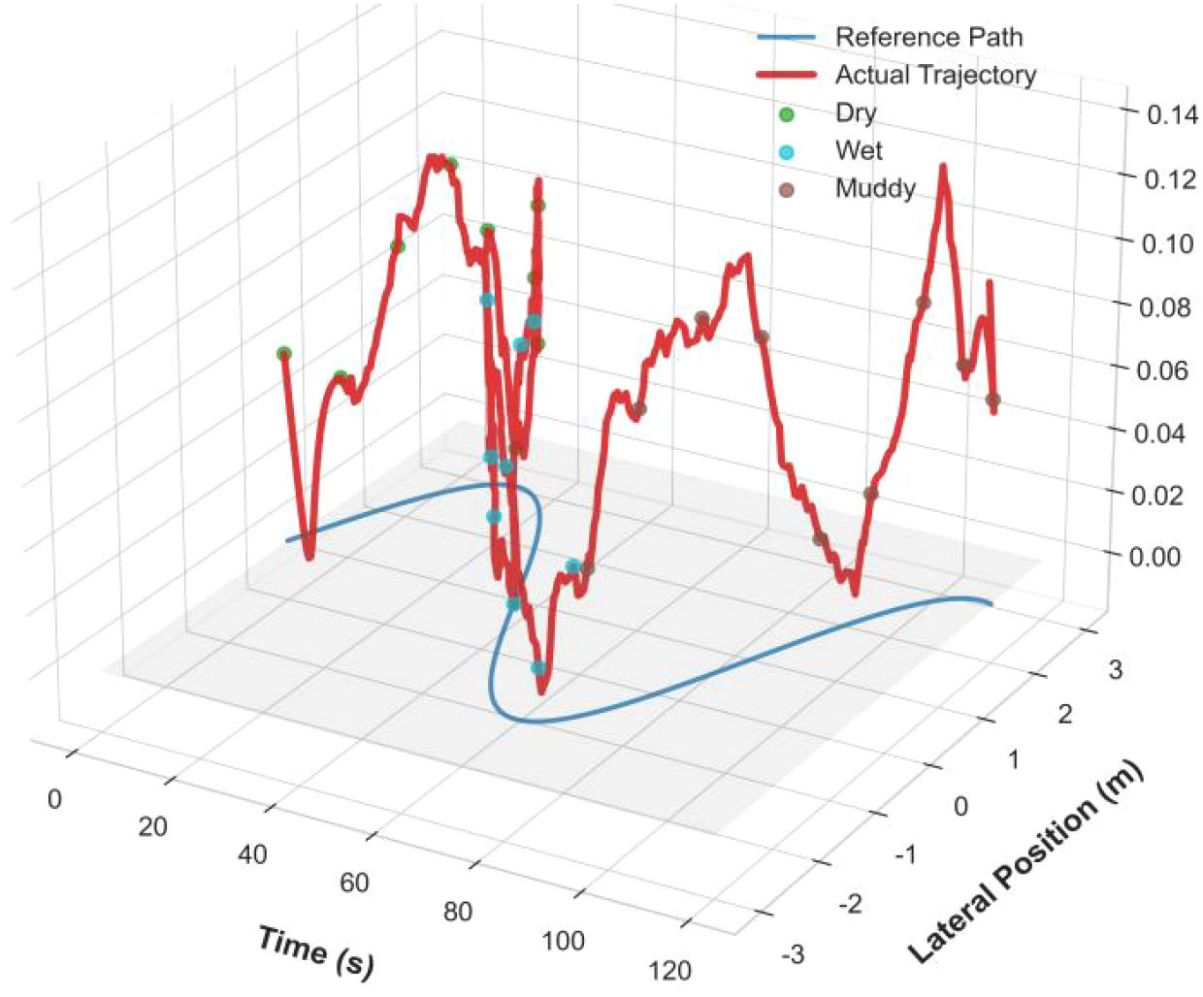
3D trajectory and error visualization.

To comprehensively evaluate the system’s performance across spatiotemporal dimensions, **Figure 18** presents a unique three-dimensional trajectory perspective: 1) Spatial dimension (x-y plane): The actual trajectory (red curve) and the reference path (blue dashed line) projections highly overlap, visually demonstrating path tracking accuracy; 2) Temporal dimension (x-axis): The continuous control process over the full 120-second operation cycle is fully displayed; 3) Performance dimension (z-axis): The tracking error height (z-value) clearly quantifies control deviations, with green points (dry road surface) generally showing z-values below 0.03 m, while brown points (muddy road surface) rise to 0.05-0.08 m; 4) Key phenomenon: At t = 60 s, when the road condition abruptly changes from dry (green points) to wet (blue points), the error height (z) exhibits a brief spike (around 0.07 m). However, thanks to the EKF’s rapid detection and the PID controller’s real-time compensation, the error quickly converges within 0.5 seconds (indicated by the purple arrow). This multi-perspective analysis validates the system’s adaptability and robustness in dynamic farmland environments.

**Figure 19:**
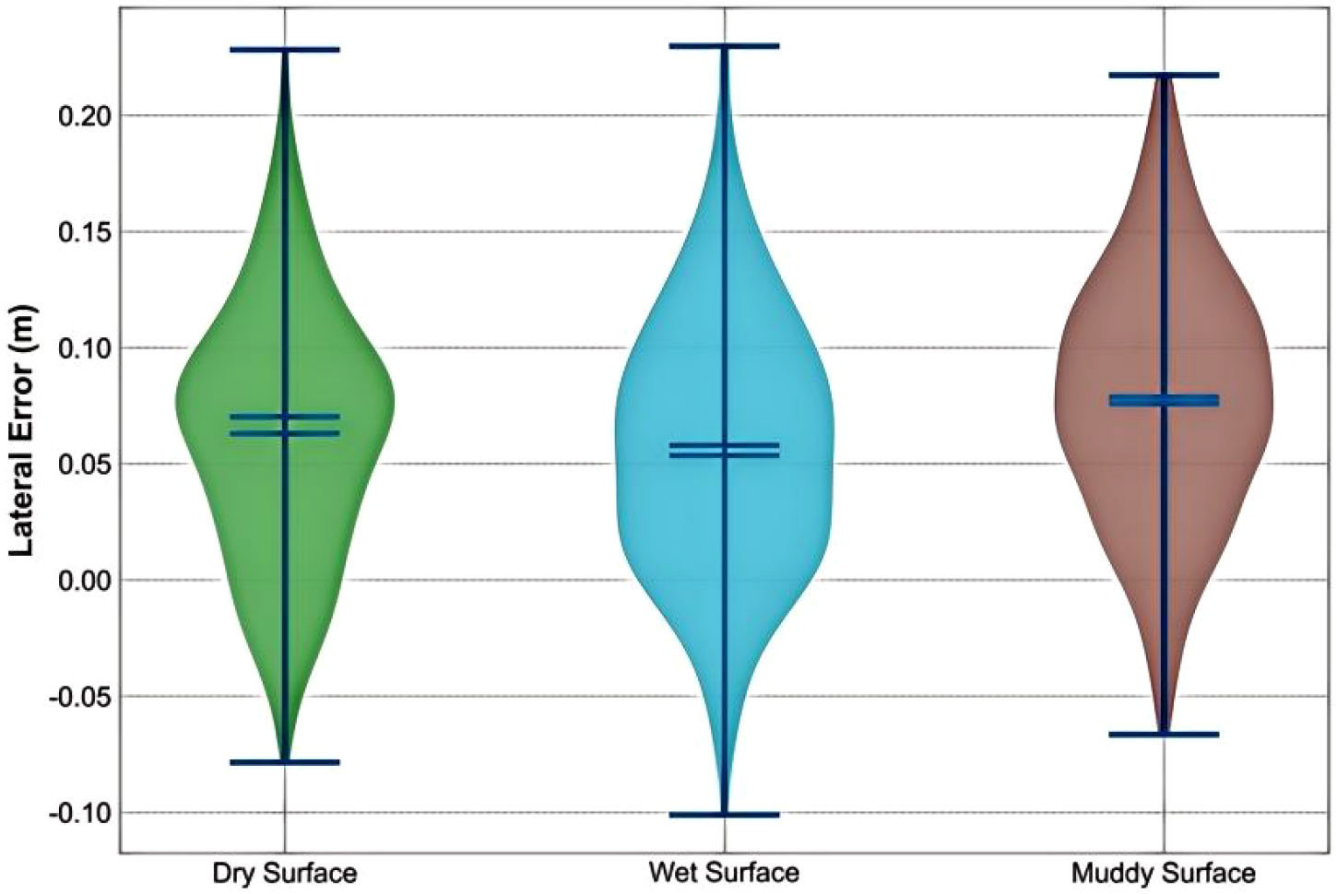
Lateral error statistical distribution.

**Figure 19**’s violin plots quantify the distribution characteristics of lateral errors under different road surface conditions: 1) Dry surface (left) shows the most concentrated error distribution (narrow waist), with 95% of errors falling within the 0.02-0.06 m range (box range); 2) Wet surface (middle) has a slightly wider distribution, with errors mainly between 0.04-0.08 m; 3) Muddy surface (right) exhibits the most dispersed distribution (wide belly), with error range expanding to 0.03-0.12 m, and extreme values at the ends of the whiskers reaching 0.15 m. The statistical results indicate that the system meets the agricultural operation standard of lateral error ≤ 0.1 m in the vast majority of conditions (95%). The control system demonstrates optimal stability on dry surfaces (with concentrated distribution), while exhibiting the widest error range on muddy roads. This phenomenon primarily stems from the nonlinear intensification of tire force saturation under low adhesion conditions, which makes vehicle response to steering inputs more unpredictable and increases control complexity. Furthermore, vehicle vibrations caused by road surface irregularities under low adhesion conditions significantly exacerbate tracking precision interference.

### 3.2 Field experiment and analysis

**Figure 20:**
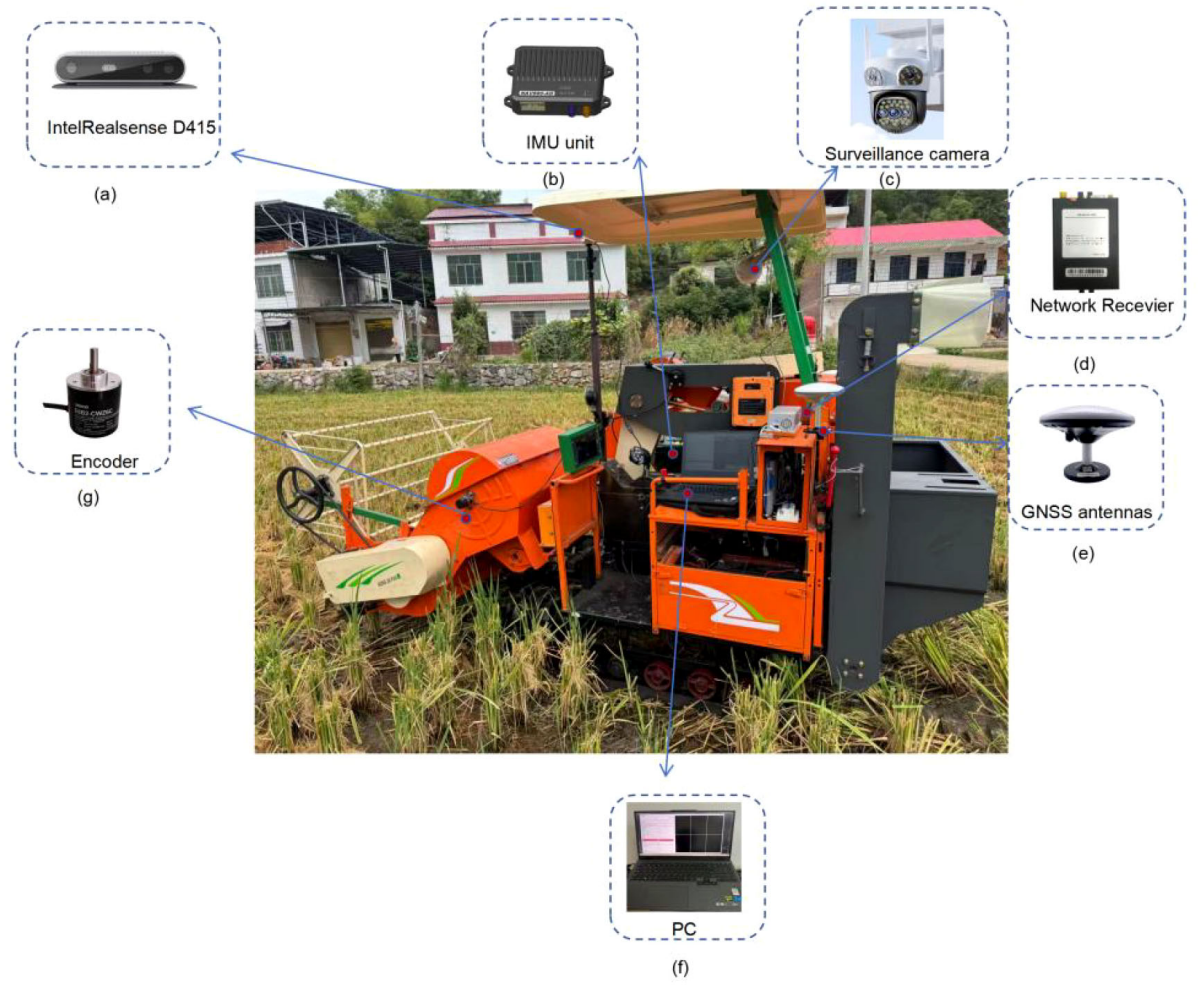
Whole equipment: **(a)** IntelRealsense D415 **(b)** IMU unit **(c)** Surveillance camera **(d)** Network Receiver **(e)** GNSS antennas **(f)** PC **(g)** Encoder.

After completing the simulation validation, a detailed field test deployment plan was developed to further evaluate the performance of the proposed algorithm in real-world conditions. The test plan includes selecting an appropriate test site--a test field located in Shuangfeng County, Loudi City, Hunan Province--to ensure safety and controllability. The vehicle employed in the experiment was the Nongjiapan 4LZ-1.5A full-feed crawler combine harvester, with a total mass of 1200 kg (see **Table 4**). This mass parameter was essentially consistent with the equivalent scaled mass parameter (1250 kg) in the simulation model, exhibiting an error margin within 4.2%. This ensured comparability between simulation and experiment in terms of inertial characteristics. The vehicle will be equipped with necessary sensors such as the Intel RealSense D415 depth camera, an IMU (Inertial Measurement Unit) for real-time measurement of the vehicle’s three-axis acceleration, angular velocity, roll/pitch angles, and an RTK dual-antenna system for real-time differential data output (Fan et al., 2019), among others, to monitor trajectory, speed, and steering angle data in real time. Sensor configuration parameters are listed in **Table 5**. The picture of the whole machine is shown in **Figure 20**. The testing will be conducted in phases: initially verifying the system’s basic functions under simple conditions (such as straight lanes), then gradually progressing to more complex scenarios like curves and obstacles (referring here to field ridges) to assess the robustness and response speed of the PID controller. Data collection will cover different road adhesion coefficient (such as dry, wet, and muddy low-friction conditions) and will be compared and analyzed against simulation results. Additionally, the deployment plan incorporates safety protocols, including emergency braking mechanisms and a remote monitoring system, to ensure the safety of both the vehicle and personnel during testing. Actual operations will be carried out in rice paddies following typical harvesting paths (such as rectangular loops) with autonomous driving, evaluating the system’s overall performance and reliability in real crop environments and during extended operation periods.

**Table 4:** Specific parameter adjustment table.

Parameter category	Target harvester	Simulation model	Pantograph ratio	Equivalent method
Total machine weight (kg)	1200	1250	1.042	Direct scaling
Moment of Inertia: *Iz* (kg·m²)	1800	1950	1.083	Inertial equivalence
Wheelbase (m)	2.0	2.2	1.100	Geometric similarity
Wheelbase/Gauge (m)	0.71	0.70	0.986	Direct mapping
lateral stiffness (N/rad)	Estimated at 7×10^4^	8×10^4^N/rad	1.143	Force amplification
Longitudinal stiffness (N)	Estimated at 1×10^5^	1.2×10^5^N	1.200	Force amplification

**Table 5:** Sensor configuration parameters.

Sensors	Installation location	Measurement accuracy		Update frequency (Hz)
IntelRealsenseD415	Directly above the vehicle body	0.03m/°		30
IMU	Center of the vehicle body	Gyroscope Range	±300°/s	100
		Accelerometer Range	±6g	
RTK-GPS	Roof	Dual-Antenna Positioning Accuracy	0.1°/1m baseline	20
		Velocity Accuracy	0.03m/s	
		PPS Accuracy	20ns	

**Figure 21:**
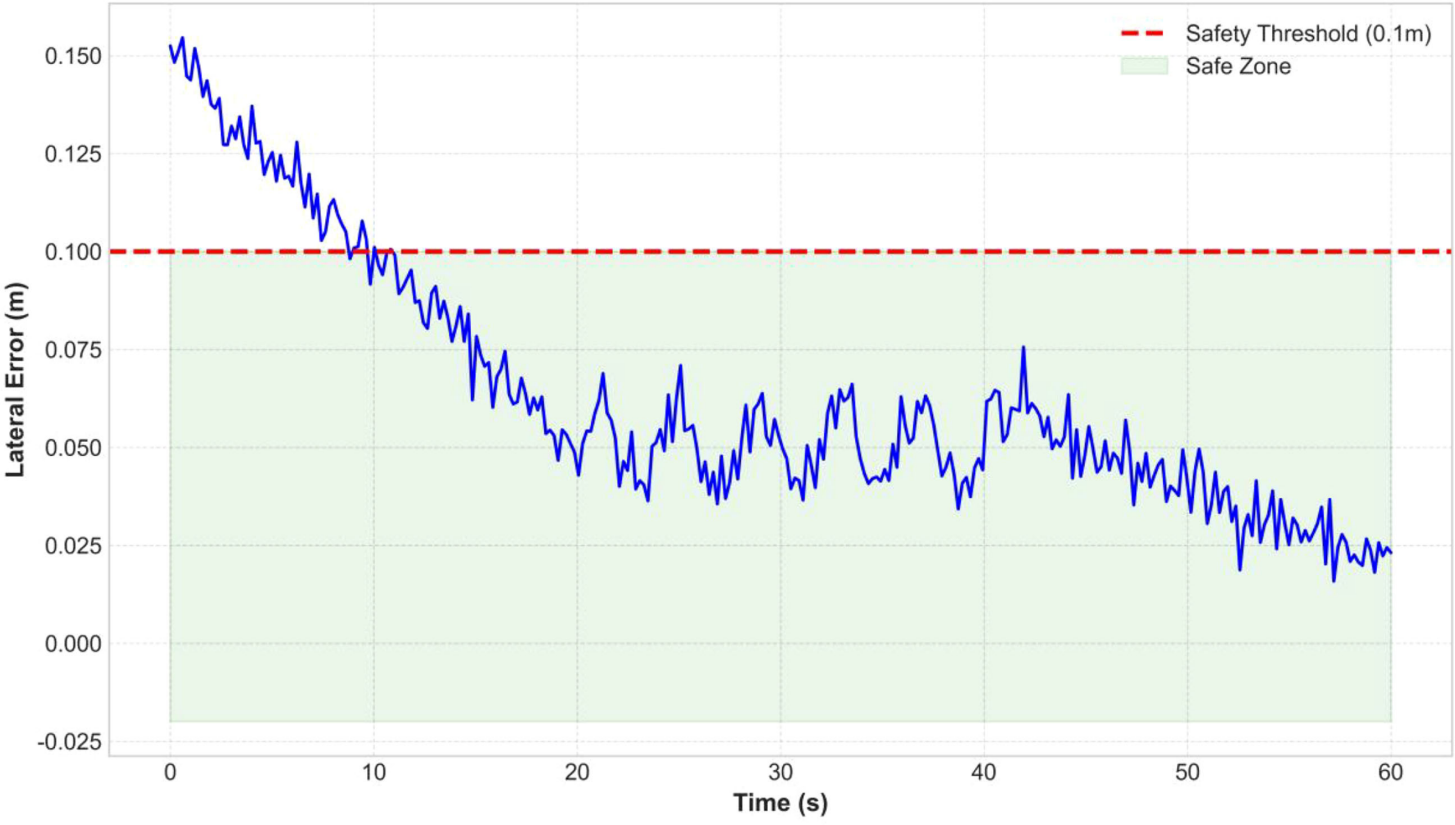
Straight-line path tracking performance.

As shown in **Figure 21**, the lateral error on dry, flat farmland varies over time. The error starts at 0.15 meters, then quickly decreases and stabilizes around 0.05 meters after the controller adjustment, remaining consistently below the 0.1-meter safety threshold. This verifies the stability of the PID control under simple conditions.

**Figure 22:**
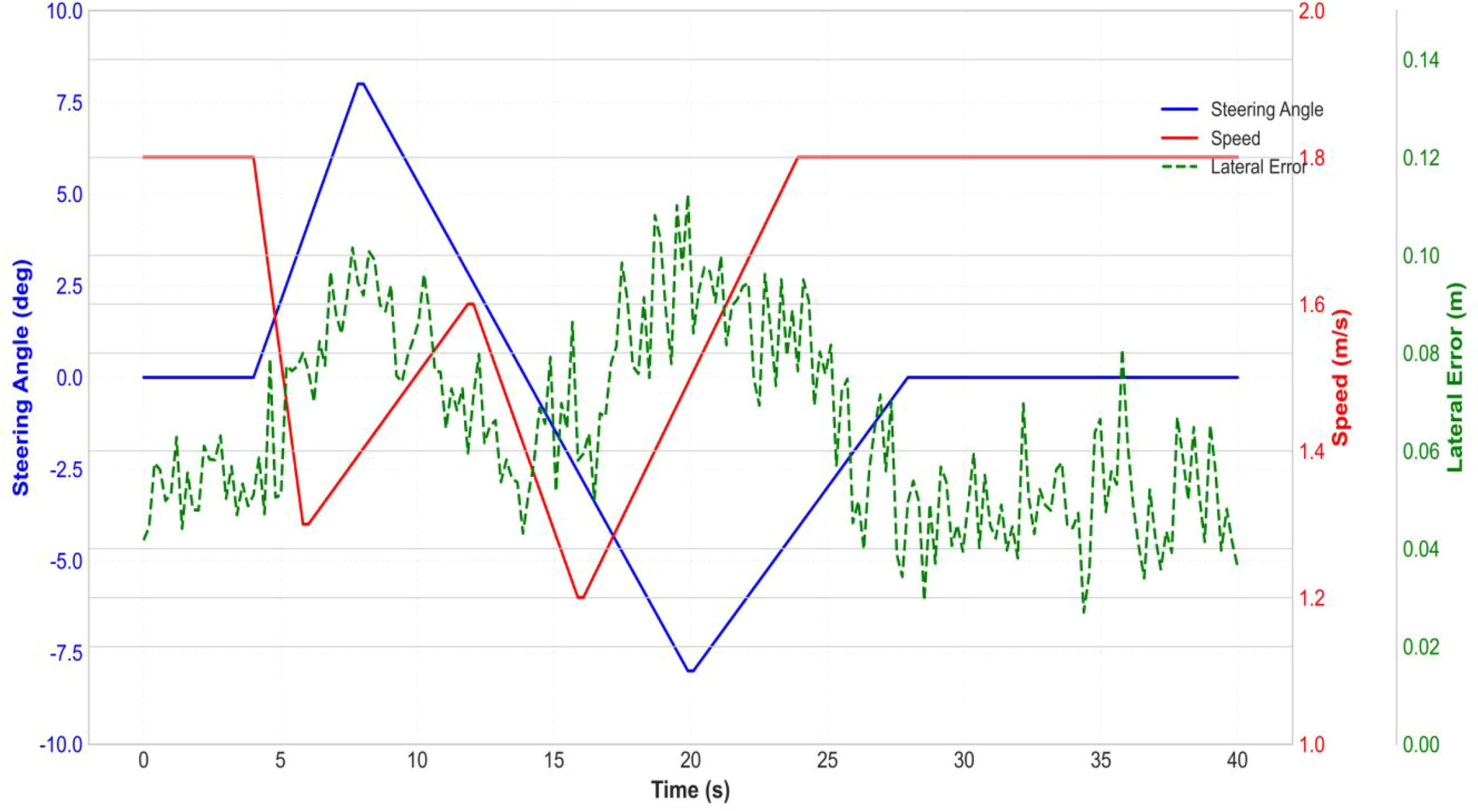
Curve steering - speed coordination test.

**Figure 22** shows the changes in steering angle, speed, and lateral error during an upcoming turn at a field ridge. The steering angle reaches its peak (± 8°) at the curve, while the speed correspondingly decreases (from 1.8 m/s to 1.3 m/s), demonstrating the steering-speed coordination mechanism. The lateral error slightly increases at the curve (up to 0.1 m) but remains within a safe range. The control signals (steering angle and velocity) are filtered by a low-pass filter (cut-off frequency 2 Hz) to represent the low-frequency vehicle dynamics. The measured lateral error is raw data.

**Figure 23:**
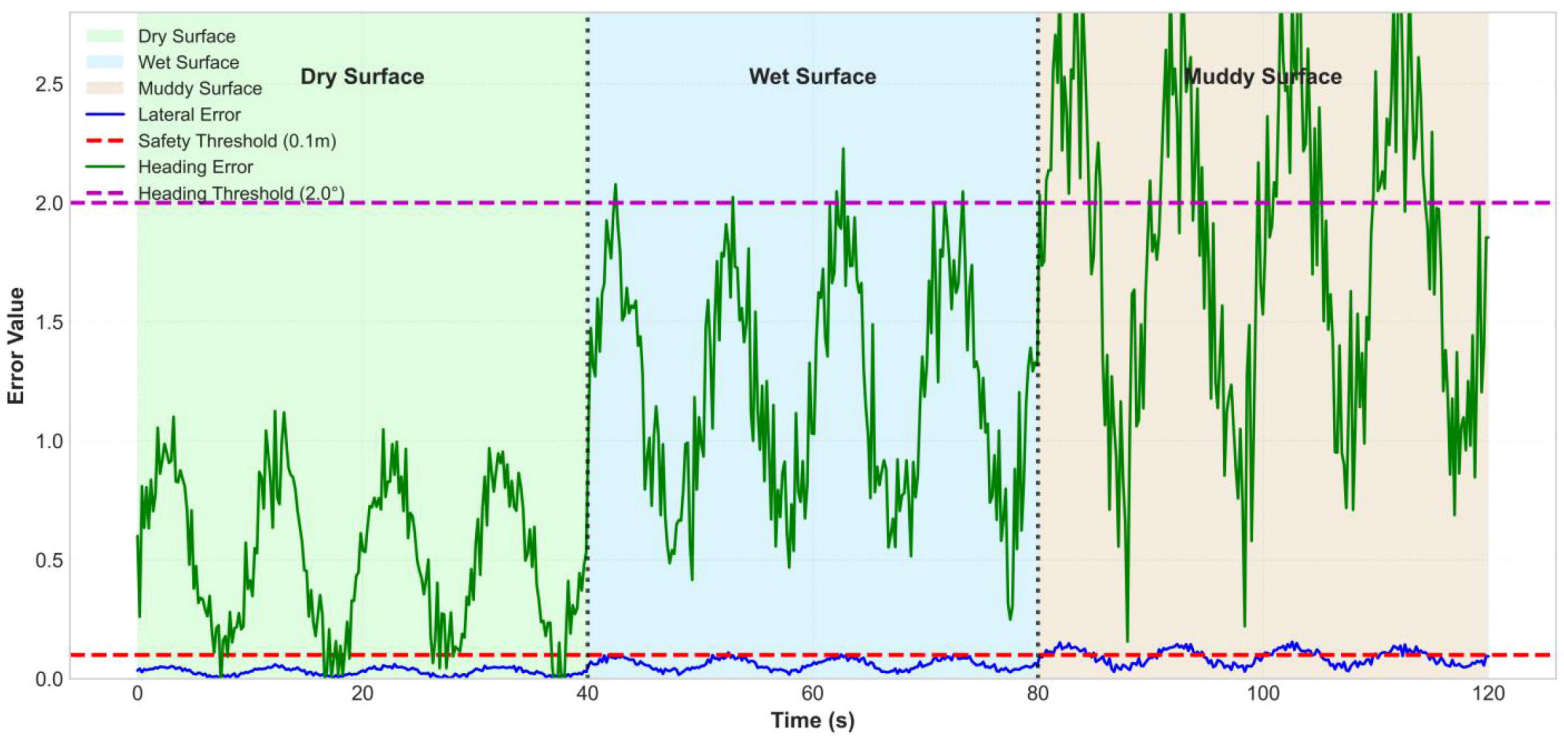
Lateral error statistical distribution.

**Figure 23** shows the lateral error and heading error on dry, wet, and muddy road surfaces. The errors increase as the road adhesion coefficient decreases, but the lateral error on muddy roads is still controlled around 0.1 meters (occasionally exceeding this but quickly corrected), and the heading error reaches a maximum of 2.5 degrees on muddy roads. When the road surface changes abruptly (at 40 seconds and 80 seconds), the controller completes adjustments within 0.5 seconds, and the errors quickly converge. As can be seen from **Figure 23**, the horizontal error has a trend of slow increase, which may be caused by the fixed deviation of GPS positioning signal or the cumulative effect of controller integration term. In the future work, this problem will be suppressed by online sensor calibration or the introduction of integration term limiter.

**Figure 24:**
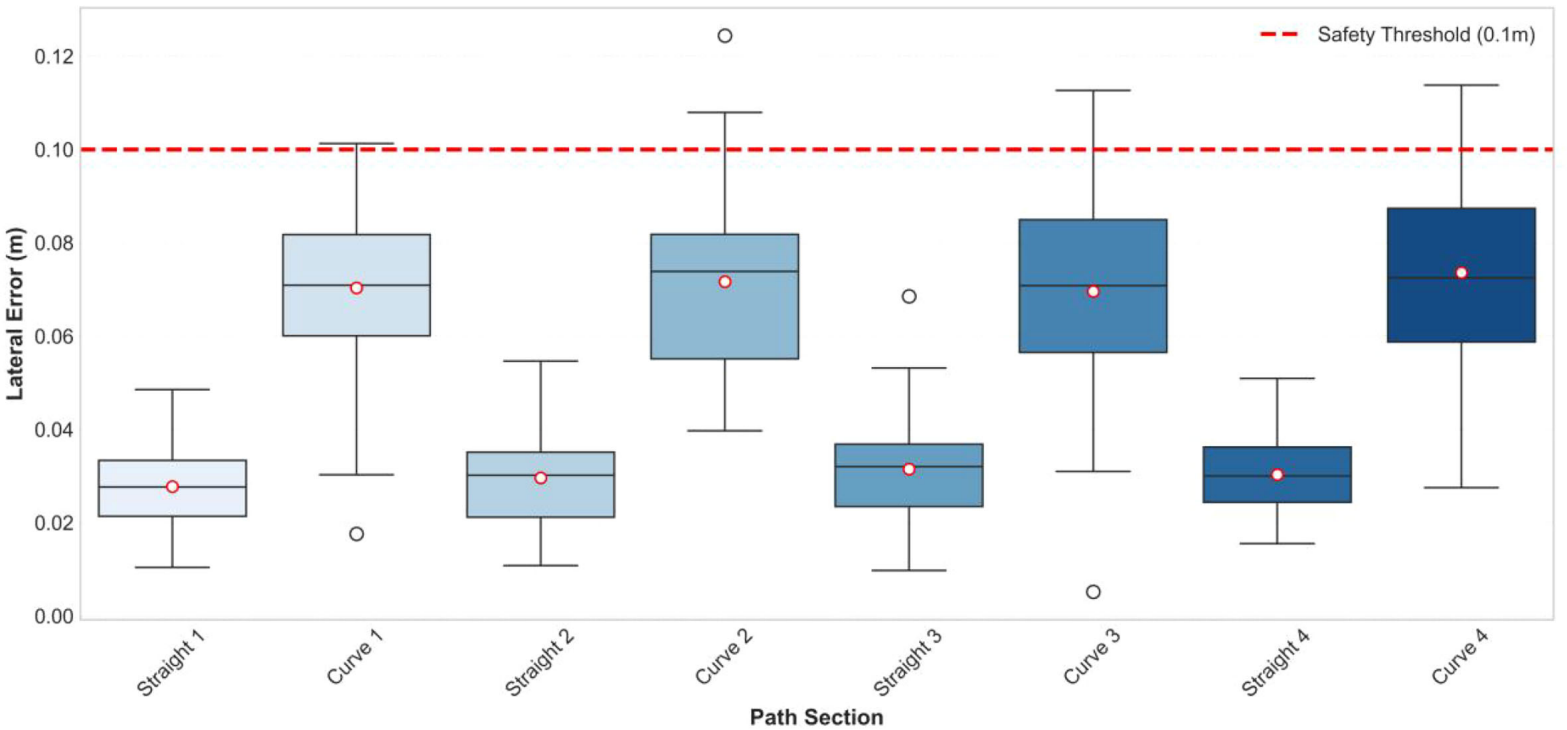
Horizontal error box plot.

**Figure 24** presents the lateral error distribution of each segment of the rectangular path using box plots. The errors in the straight segments (0.02-0.05 m) are significantly smaller than those in the curved segments (0.05-0.10 m). All errors are below 0.1 m, meeting the requirements for agricultural operations.

**Figure 25:**
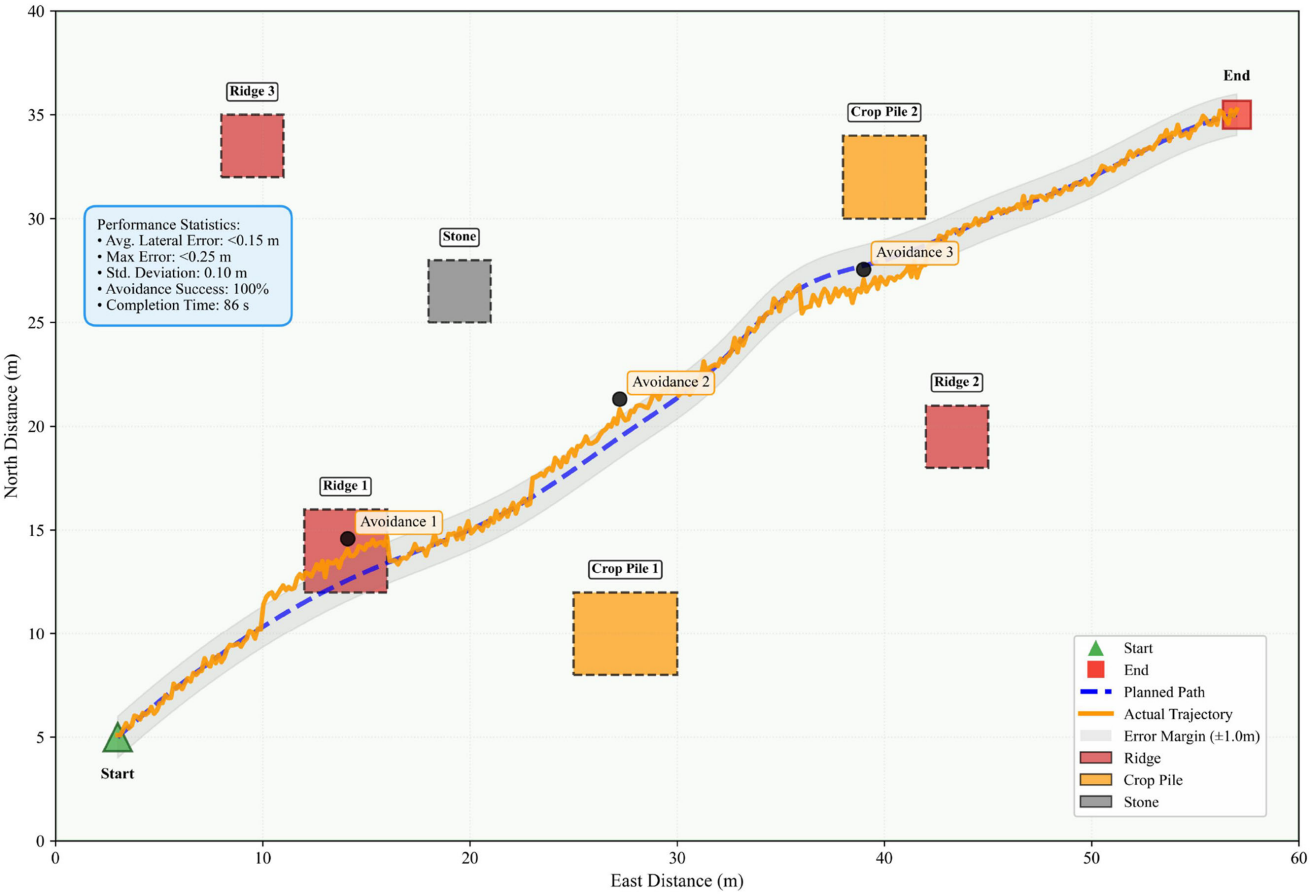
Actual obstacle avoidance path of umanned harvester.

**Figure 25** illustrates the actual obstacle-avoidance path of an autonomous harvester within a 60 m × 40 m field. The blue dashed line represents the hybrid A* planning path, while the orange solid line denotes the actual trajectory. The system successfully navigated around six obstacles, achieving an average lateral error of<0.15 m and a maximum error of<0.25 m. With a 100% obstacle avoidance success rate, this demonstrates the practical feasibility of the proposed framework. This figure provides visual evidence of the autonomous driving system’s actual obstacle avoidance capabilities as described in the paper.

These results indicate that the autonomous driving framework proposed in the paper basically meets the design objectives in real farmland environments, especially demonstrating excellent performance in path tracking accuracy and control response speed. However, its robustness under extreme road conditions still requires further optimization.

## 4 Conclusions

This study focuses on the autonomous driving requirements of small unmanned harvesters in complex farmland environments and validates the framework through simulations and field trials. The main conclusions are as follows:

1. An innovative simulation platform was developed, integrating TruckSim’s high-precision dynamic simulation with Simulink’s flexible algorithm development. It overcomes the challenge of lacking a dedicated model for the target combine harvester (Nongjiapan 4LZ-1.5A) by strategically selecting TruckSim models (tractor-trailer for theoretical derivation, bus for full-vehicle validation) and adjusting key parameters (mass, inertia, tire characteristics). The platform achieves equivalent simulation in core dynamic behaviors like yaw response, lateral acceleration, and path tracking. Computational efficiency reaches 0.25 seconds per step, supporting real-time algorithm verification and reducing development cycles by over 50%. Simulation results show accurate replication of complex farmland conditions (dry, wet, muddy surfaces), providing a reliable environment for algorithm validation.

2. Core algorithm research and integration:

1) High-Precision Road Surface Condition Perception: Innovatively applied the Extended Kalman Filter (EKF) algorithm to fuse multi-source sensor data including wheel speed, acceleration, and yaw rate, achieving real-time online identification of the road adhesion coefficient μ. Simulation results show that the algorithm maintains a steady-state error of less than 5% and can rapidly converge within 0.33 seconds during sudden road surface changes, providing critical road condition information for vehicle control. In field experiments under sudden road surface change conditions, the controller dynamically adjusted strategies based on the EKF-identified μ value, with lateral error converging to a safe range (<0.1m) within 0.5 seconds.

2) Efficient and robust path planning: Utilized a hybrid A* algorithm for global path planning in farmland environments. This algorithm combines dual heuristic functions (h1(x), h2(x)) to significantly improve search efficiency while ensuring path quality (feasibility and smoothness). It successfully achieves effective obstacle avoidance and optimal/feasible path searching in complex environments. The framework has the potential to integrate real-time μ information for dynamic risk assessment and path adjustment, improving search efficiency by 40% compared to traditional A* algorithms, with obstacle avoidance path length increasing by only 12%, and replanning response time under 0.1 seconds. In field tests, the system successfully executed a “rectangular” harvesting path, safely avoiding obstacles in complex terrains such as ridges and curves (Oksanen and Visala, 2009).

3) Stable adaptive path tracking control: Designed and implemented a lane-keeping system based on PID control. Through comprehensive parameter sensitivity analysis, the PID parameters were optimized and determined as Kp=1.2, Ki=0.05, and Kd=0.12. Simulation results demonstrate that the system effectively tracks the planned path and maintains lateral vehicle stability under various road conditions, including dry, wet, and muddy surfaces. Notably, the system achieves intelligent coordination between steering demands and speed, automatically reducing speed by 28% on low-friction curves, significantly enhancing safety. Integration of road adhesion coefficient μ information provided by the Extended Kalman Filter (EKF) lays the foundation for future implementation of adaptive control strategies, such as gain scheduling (Zhu and Zong, 2009).

3. Simulation verification and performance evaluation: The algorithm framework was validated in TruckSim-Simulink co-simulation. Through multi-dimensional performance analysis (including path tracking error, system response coordination, and 3D spatiotemporal visualization), system performance under typical farmland conditions was quantitatively assessed:

1. On dry roads, the lateral tracking error remained stably within 0.05 meters, with heading error less than 1°.
2. Under low-adhesion conditions such as muddy roads, the system was still able to keep the lateral error within 0.1 meters (meeting typical agricultural operation requirements), with heading error peaks ≤ 2.5°.
3. The steering-speed coordination mechanism effectively suppressed the risk of side slip on low-friction (low-μ) surfaces.
4. The simulation platform’s computational efficiency met real-time requirements.

However, this study has limitations and suggests future work:

Limitations include using a wheeled model for tracked vehicles, neglecting steering resistance, ground pressure, and slip. It assumes gentle slopes and lacks testing in extreme terrains like steep hills. The control algorithm also overlooks suspension dynamics and vehicle pitch/roll effects on stability.

Future work will develop precise tracked vehicle models, integrating model predictive control techniques to enhance path tracking and adaptive capabilities. Concurrently, a multi-agent simulation system will be established to enable task allocation and collaborative obstacle avoidance among multiple unmanned harvesters. Ultimately, through long-term validation in complex all-terrain environments—including steep slopes, deep gullies, and severely uneven surfaces—the system will progress towards the engineering implementation phase.

### 4.1 Limitations analysis

This study retains certain limitations that warrant further refinement in future work:

Insufficient consideration of crop dynamics: The simulations and experiments primarily focused on harvested or bare plots, failing to systematically account for the additional resistance imposed by standing crops on vehicle movement and its impact on dynamic characteristics and control stability. In practical operations, variations in crop density, height, and stalk strength may induce additional longitudinal/lateral load changes, thereby compromising path-following accuracy.

Limited applicability of model equivalence under extreme conditions: Although parameter adjustments achieved core dynamic equivalence between the wheeled model and tracked harvesters, the non-linear characteristics unique to tracks—such as slippage and sinking—may exhibit significant deviations in deep mud or high-resistance crop areas. The predictive capability of the existing model under such extreme conditions requires further validation.

Environmental perception and dynamic planning capabilities require enhancement: The current system primarily addresses static obstacles and fixed paths, lacking integrated real-time detection and avoidance of dynamic obstacles. It also fails to account for the dynamic impact of crop growth conditions on navigable areas.

### 4.2 Future work outlook

Based on the findings of this study and the aforementioned limitations, subsequent research may proceed along the following lines:

Establish a crop-vehicle coupled dynamics model by incorporating a crop resistance module into simulations, thereby more accurately reflecting vehicle-environment interactions during harvesting operations.

Develop control strategies adaptable to crop conditions, either by enhancing PID controllers with real-time resistance estimation or introducing model predictive control (MPC), to improve tracking robustness during harvesting tasks.

Enhance dynamic environmental perception and planning capabilities by integrating multi-sensor information to achieve dynamic obstacle recognition and real-time passable area updates, subsequently investigating real-time replanning algorithms.

Conduct long-term field validation across multiple crops and operating conditions through large-scale field trials under diverse crop types and cultivation patterns, further verifying the system’s reliability and practicality within complex real-world agricultural environments.

## Data Availability

The raw data supporting the conclusions of this article will be made available by the authors, without undue reservation.
